# Examining the Efficacy of the Telehealth Assessment and Skill-Building Kit (TASK III) Intervention for Stroke Caregivers: Protocol for a Randomized Controlled Clinical Trial

**DOI:** 10.2196/67219

**Published:** 2025-03-25

**Authors:** Tamilyn Bakas, Elaine Miller, Heidi Sucharew, Natalie Kreitzer, Jahmeel Israel, Matthew Rota, Brett Harnett, Kari Dunning, Holly Jones, Michael McCarthy, Bonnie Brehm, Joan K Austin, Pamela H Mitchell

**Affiliations:** 1 Department of Population Health College of Nursing University of Cincinnati Cincinnati, OH United States; 2 Department of Emergency Medicine College of Medicine University of Cincinnati Cincinnati, OH United States; 3 Department of Biomedical Informatics College of Medicine University of Cincinnati Cincinnati, OH United States; 4 Department of Rehabilitation Exercise and Nutrition Sciences College of Allied Health Sciences University of Cincinnati Cincinnati, OH United States; 5 Martha S. Pitzer Center for Women, Children, and Youth College of Nursing The Ohio State University Columbus, OH United States; 6 Department of Social Work Northern Arizona University Flagstaff, AZ United States; 7 Center for Enhancing Quality of Life in Chronic Illness School of Nursing Indiana University Indianapolis, IN United States; 8 Department of Biobehavioral Nursing and Health Systems University of Washington Seattle, WA United States

**Keywords:** stroke, family caregivers, depressive symptoms, health-related quality of life, clinical trial, intervention study, protocol, nursing

## Abstract

**Background:**

Stroke is a leading cause of serious, long-term disability and has a sudden onset. Upon discharge to the home setting, families are thrust into providing care, often without sufficient training from health care providers. Aligned with current patient and caregiver guidelines, the Telehealth Assessment and Skill-Building Kit (TASK III) is a nurse-led intervention designed to empower caregivers to address their own needs and those of the survivor using innovative skill-building strategies.

**Objective:**

This study aims to test the short-term (immediately after the intervention at 8 wk) and long-term (12, 24, and 52 wk) efficacy of the TASK III intervention, compared with an information, support, and referral (ISR) group, to improve caregiver life changes (ie, changes in physical health, physical functioning, emotional well-being, and general health) as a result of providing care.

**Methods:**

A randomized controlled clinical trial design will be used with baseline data collection from 296 family caregivers by telephone after the stroke survivor is discharged home. Caregivers randomly assigned to the ISR group (n=148, 50%) will receive information from the American Heart Association about stroke family caregiving. Caregivers randomly assigned to the TASK III group (n=148, 50%) will receive a TASK III resource guide and information from the American Heart Association. Both groups will receive 8 weekly calls from a nurse, with a booster call a month later. Outcomes will be assessed by blinded data collectors at 8, 12, 24, and 52 weeks. The primary outcome (at 8 wk) is caregiver life changes measured by the Bakas Caregiving Outcomes Scale. Secondary outcomes are depressive symptoms; other symptoms (eg, stress, fatigue, sleep, pain, and shortness of breath); unhealthy days; diet; exercise; and self-reported health care use. Mediators are task difficulty, threat appraisal, and self-efficacy. Program evaluation outcomes (satisfaction and technology ratings) will also be analyzed.

**Results:**

The trial was registered on March 10, 2022. Enrollment and random assignment of the first participant was on November 30, 2022, with an anticipated completion of recruitment by November 30, 2025. Completion of the primary end point data analysis is anticipated by August 31, 2026, with results expected to be reported on ClinicalTrials.gov by April 1, 2027. As of October 9, 2024, a total of 198 (66.9% of the proposed total sample of 296) family caregivers have been enrolled and randomly assigned to the TASK III group (n=98, 49.5%) or the ISR group (n=100, 50.5%). The last update was performed on January 25, 2024.

**Conclusions:**

If the TASK III intervention is shown to be efficacious in the proposed randomized controlled clinical trial, our next goal will be to translate TASK III into ongoing stroke systems of care, providing a tremendous public health impact.

**Trial Registration:**

ClinicalTrials.gov NCT05304078; https://clinicaltrials.gov/study/NCT05304078

**International Registered Report Identifier (IRRID):**

DERR1-10.2196/67219

## Introduction

### Background and Rationale

Each year, approximately 795,000 Americans experience a stroke, which is a leading cause of serious, long-term disability [1]. Because of disability, approximately 68% to 74% of stroke survivors require the care of family members [2,3], who are suddenly thrust into providing care without receiving proper training from health care providers [4-6]. The lack of training contributes to difficulty with caregiving tasks and high threat appraisal, defined as caregiver perceptions of ability to provide future care [7-10]. These stressful experiences place caregivers at risk for depressive symptoms and major life changes as a result of providing care (eg, physical health, physical functioning, emotional well-being, and general health) [7-15]. Finally, stressful caregiver experiences have been reported to impede the survivor’s rehabilitation [16-18] and to increase costs from premature long-term institutionalization [11,19,20].

Major scientific and policy statements from the American Heart Association [17,21-25] and other guidelines [26-30] recommend (1) assessment of stroke family caregiver needs and concerns; (2) education on stroke-related care; (3) attention directed toward caregivers’ physical and emotional health; and (4) individualized caregiver interventions that combine psychoeducational strategies with skill building (eg, problem-solving, stress management, and goal setting). Aligned with the American Heart Association recommendations and other published guidelines [17,21-30], the Telephone Assessment and Skill-Building Kit (TASK) II is a nurse-led intervention enabling caregivers to build skills based on the assessment of their own needs [31-38]. Unlike existing stroke caregiver interventions that require costly face-to-face interactions [22,23,33,39-41] and that focus primarily on the survivor’s care [22,23,39-41], TASK II is delivered completely via telephone and empowers caregivers to address both their own and the survivor’s needs using innovative skill-building strategies (eg, problem-solving, stress management, realistic expectations, time management, and communication with health care providers) using a mailed TASK II resource guide and calls with a nurse. Evidence has been published regarding content validity [32], treatment fidelity [36], caregiver satisfaction [32], and efficacy of the TASK II program with 254 stroke caregivers in a randomized controlled clinical trial (R01; R01NR010388; ClinicalTrials.gov ID: NCT01275495) [34]. TASK II, in comparison with an information, support, and referral (ISR) group, reduced depressive symptoms up to a year after baseline (in caregivers with mild to severe depressive symptoms) and reduced unhealthy days, a reflection of caregiver’s physical and mental health at 8 weeks [34]; however, further enhancements were needed to improve caregiver life changes (ie, physical health, physical functioning, emotional well-being, and general health) and to provide additional telehealth modes of delivery based on caregiver preferences [34,38,42-44].

Through a funded R21 (R21NR016992; ClinicalTrials.gov ID: NCT03635151) feasibility study, the Telehealth Assessment and Skill-Building Kit (TASK III) version has been optimized for delivery via various modes of technology [38] and provides a stronger emphasis on caregiver self-management [37,45,46] of their own symptoms and health care needs to sustain positive life changes and health outcomes over time. Caregivers can now choose how they want to access the TASK III resource guide (mailed hard copy, e-book, USB drive, or website) and how they would like to interact with the nurse (telephone, FaceTime, or web-based videoconferencing). Our published findings demonstrate the usefulness, ease of use, convenience, feasibility, and acceptability of our new TASK III telehealth technology [38]. Our published findings have also shown content validity, accuracy, feasibility, and acceptability of our new skill-building tip sheet on goal setting to enhance caregiver self-management of symptoms and health [37]. As part of the R21 feasibility study, a pilot study with 74 stroke caregivers randomly assigned to the TASK III (n=36) or ISR (n=38) group revealed successful recruitment, retention, treatment fidelity, high satisfaction ratings, and positive data trends.

In this larger randomized controlled clinical trial (R01NR020184; ClinicalTrials.gov ID: NCT05304078), we plan to enroll 296 caregivers to test the efficacy of the TASK III program (n=148, 50%), in comparison with the ISR group (n=148, 50%) for improving stroke caregiver life changes, symptoms, and health outcomes. The TASK III group will receive the TASK III resource guide along with information from the American Heart Association about stroke family caregiving. The ISR (attention control) group will only receive information from the American Heart Association. Both groups will receive 8 weekly calls from a nurse with a booster call a month later. The nurse calls for the TASK III group to focus on assessing and addressing stroke caregiver needs and concerns using the TASK III resource guide, along with information from the American Heart Association; support through active listening; and provide referrals to their own health care providers. The nurse calls for the ISR comparison group to focus only on information from the American Heart Association, support through active listening, and provide referrals to their own health care providers. The ISR group procedures have been used successfully as a comparator for our previous trials (eg, R01 and R21). Data will be collected at baseline and at 8, 12, 24, and 52 weeks after randomization.

### Objectives

#### Specific Aim 1

This study aims to test the short-term (immediately after the intervention at 8 wk) and long-term (12, 24, and 52 wk) efficacy of the TASK III intervention, compared with the ISR group, in improving the *primary outcome* of caregiver life changes (ie, physical health, physical functioning, emotional well-being, and general health) as a result of providing care.

#### Specific Aim 2

This study also aims to test the short-term (immediately after the intervention at 8 wk) and long-term (12, 24, and 52 wk) efficacy of the TASK III intervention, compared with the ISR group, in improving *secondary outcomes* including caregiver depressive symptoms (in caregivers with mild to severe depressive symptoms); other symptoms (stress, fatigue, sleep, pain, and shortness of breath); unhealthy days; self-management of diet and exercise; self-reported health care use; *mediators* (caregiver task difficulty, threat appraisal, and self-efficacy for exercise and diet); and *program evaluation outcomes* at 12 weeks (caregiver satisfaction and technology ratings).

## Methods

### Trial Design

**Figure 1:**
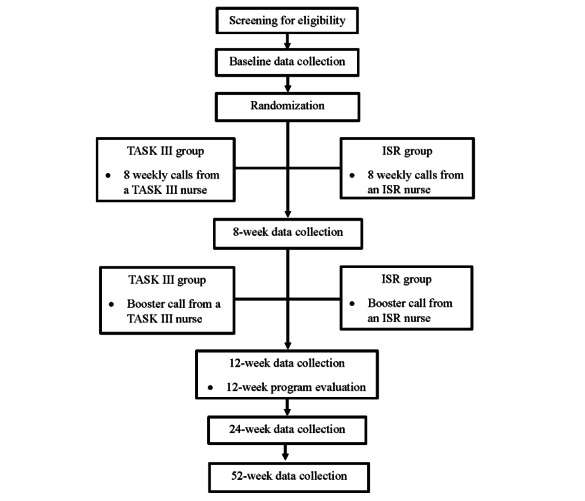
Study flow diagram. ISR: information, support, and referral; TASK III: Telehealth Assessment and Skill-Building Kit.

An experimental, 2-group, randomized controlled clinical trial design based on a superiority framework will be used to test the efficacy of the TASK III program compared with an ISR group using an intention-to-treat design. We will randomly assign 296 stroke caregivers using a 1:1 allocation ratio to either the TASK III intervention group (n=148, 50%) or the ISR attention control group (n=148, 50%) following baseline data collection by telephone after the stroke survivor is discharged home (Figure 1). Follow-up data collection by telephone will occur by blinded data collectors after the 8 weekly TASK III or ISR calls, after the booster call at 12 weeks, and then at 24 and 52 weeks. Program evaluation by telephone will occur at 12 weeks after all the calls from the nurse have been completed while maintaining blinding of the data collectors.

### Participants, Interventions, and Outcomes

#### Study Setting

Data will be collected from family caregivers living in the community caring for stroke survivors after discharge to the home setting. Recruitment sites are acute care and rehabilitation hospitals that provide care for stroke survivors located in the United States Midwest region. A list of our recruitment sites as of October 7, 2024, is provided in Multimedia Appendix 1. Further details regarding recruitment are provided in the *Recruitment* section.

#### Eligibility Criteria

Inclusion and exclusion criteria for stroke family caregivers are summarized in Textbox 1.

Eligibility criteria for the nurse interveners for both the TASK III and ISR groups include having a registered nurse license; strong interpersonal skills; and proficiency with technology, including Zoom (Zoom Communications) and Teams (Microsoft Corp) videoconferencing and basic computer skills. Blinded data collectors are required to have a high school diploma (college education preferred); strong interpersonal skills; and basic computer skills including the ability to type and enter data.

**Inclusion and exclusion criteria for stroke family caregivers.**

**Inclusion criteria**

- Primary caregiver (family member or a significant other providing care for a stroke survivor at home)
- Fluent in the English language (ie, able to read, speak, and understand English)
- Access to a telephone or computer
- No difficulties hearing or talking by a telephone or computer
- Scores ≥4 on a 6-item cognitive impairment screener
- Willing to participate in 9 calls from a nurse and 5 data collection interviews (baseline, 8, 12, 24, and 52 wk)

**Exclusion criteria if the following were true for the survivor**

- Had not had a stroke
- Did not need help from the caregiver
- Resides in a nursing home or long-term care facility

**Exclusion criteria if the following were true for the survivor or caregiver**

- Aged <18 years
- Prisoner or on house arrest
- Pregnancy
- Terminal illness (eg, late-stage cancer, end-of-life condition, or renal failure requiring dialysis)
- History of Alzheimer disease, dementia, or severe mental illness (eg, suicidal tendencies, schizophrenia, severe untreated depression, or manic depressive disorder)
- History of hospitalization for alcohol or drug abuse within the past 5 years

### Ethical Considerations

The study was approved by the University of Cincinnati Institutional Review Board (IRB; study 2022-0180). Lists of contact information for stroke survivors and next of kin will be obtained from each recruitment site. Approximately 1 week after study flyers and informed consent forms are mailed, stroke caregivers will be contacted via telephone by the TASK III study research staff to determine their interest in the study and to screen for eligibility. Flyers with response cards and informed consent forms may also be distributed by staff employed at each recruitment site.

The informed consent process will take place via telephone. The informed consent form is provided in Multimedia Appendix 2. The process will be explained to interested family caregivers, and if they are eligible and provide verbal consent, a baseline interview will be scheduled. All caregivers will be provided with full information about the nature, purpose, and possible risks and benefits of the study. Furthermore, caregivers will be informed that participation in the study is voluntary, and they are free to discontinue participation at any time. The principal investigator (PI) or research staff trained by the PI will obtain initial and ongoing consent. Training of research staff will include reading the informed consent and role-playing the consent process with the PI. All research staff having contact with the caregivers in the study will be trained on this process. The telephone calls to explain the consent will be scheduled at a time that is convenient for the caregiver using a telephone number provided by the caregiver. The PI or trained research staff will make the calls from a private location to protect the confidentiality of the caregiver. At the beginning of the baseline interview, the informed consent form will be reviewed again with caregivers, with verbal consent being verified before baseline data collection. Caregivers who are eligible, have provided verbal consent, and have completed baseline data collection will be enrolled and randomly assigned into either the TASK III or ISR group. Because this is a minimal risk study, the IRB has approved a waiver of written documentation of consent. We have previously conducted 4 IRB-approved studies using the same recruitment strategies as detailed in the current protocol [7,31,34,37,38]. Our procedures have not been found to be coercive by the IRB, and we have been successful in recruiting and retaining caregivers in our previous studies. In fact, we believe that approaching caregivers in person while the survivor is still hospitalized may be more coercive than requesting their participation by mail and telephone. Caregivers receiving their informed consent by mail approximately a week in advance have more time to read it and consider participation than if they were approached in person.

Only authorized study personnel will have access to the database or contact information for potential family caregivers, as well as family caregivers enrolled in the study. Study identification numbers will be generated to protect the identity of the participants. Participants will not be identified in any manner in reports or manuscripts from the study. Data from all data collection interviews and nurse calls will be audio recorded. Audio recordings will be uploaded to a secure password-protected OneDrive (Microsoft Corp) folder at the University of Cincinnati and then deleted from the digital recorders. Once the audio recordings are monitored for adherence to the protocol or transcribed (deidentified), they will be deleted to protect the anonymity of the participants. Data entered on paper forms will be entered into REDCap (Research Electronic Data Capture; Vanderbilt University) by trained research staff using a secure log-in. Deidentified paper forms will be stored in a locked file cabinet accessible only to study personnel or shredded once data entry into REDCap is complete. REDCap is backed up nightly by the institution. Only authorized study personnel will have access to REDCap. All data collection calls to participants will be made to a telephone number provided by the participant at a time that is convenient for the participant. The TASK III and ISR group calls will be made via telephone, FaceTime, or Zoom videoconference using a secure link that is protected by the Health Insurance Portability and Accountability Act (HIPAA) based on caregiver preference. Research staff will make calls from a private location to protect confidentiality. All research staff will be trained in procedures to protect the confidentiality of the participants, including referring to caregivers using only their study ID numbers while communicating with the PI, project manager, or other approved research staff. Procedures for tracking completed interviews, determining the appropriate dates for follow-up interviews, and providing reminders for missed interviews will be specified and tracked in REDCap.

Although no compensation is provided for this study, participants are permitted to keep any study materials mailed to them, including a pedometer, Amazon Fire Tablet, USB drive, and a hard copy binder with either the TASK III or ISR content.

### Interventions

#### Explanation for the Choice of Comparators

The comparator for this study will be the ISR program that has been used and validated as an attention control group in our previous studies testing the TASK, TASK II, and TASK III programs [31,32,34-36]. Caregivers randomly assigned to the ISR group have expressed high satisfaction ratings (ie, usefulness, ease of use, and acceptability) [32] with low attrition [31,34] and high fidelity of procedures [36]. Stroke caregivers randomly assigned to the ISR group for this study will receive a mailed copy of an American Heart Association brochure about stroke family caregiving and 8 weekly phone calls from a nurse with a booster call at 12 weeks. Calls will focus on providing ISR via the use of active listening strategies [31,32,34-36]. The ISR group can access the American Heart Association brochure through its website, which offers publicly available guidelines and web-based information for family caregivers. In addition, caregivers will receive a pedometer to track their daily steps, an Amazon Fire Tablet and a USB drive for use in the ISR program, and a unique username and password to log in to the ISR group on our TASK III website, which contains links to the American Heart Association materials for stroke family caregivers. Caregivers in the ISR group can choose to receive calls from their nurse via telephone, FaceTime, or Zoom videoconferencing. The length of each ISR session will vary based on the needs of the caregiver. As in our previous study with the TASK II program [34], our R21 feasibility trial showed that the total number of minutes on the ISR calls averaged about 17 minutes for each of the 9 ISR calls (mean 156, SD 103 min).

#### Intervention Description

Stroke caregivers randomly assigned to the TASK III intervention will receive a mailed copy of the TASK III resource guide, a mailed copy of an American Heart Association brochure about stroke family caregiving, a TASK III USB drive, and instructions for accessing a TASK III e-book and the TASK III interactive website. Screenshots of TASK III website are provided in Multimedia Appendix 3 [47].

#### TASK III Resource Guide

In a mailed binder with numbered tabs, the Caregiver Needs and Concerns Checklist (CNCC) [4] addresses 5 areas of needs: finding information about stroke, managing the survivor’s emotions and behaviors, providing physical care, providing instrumental care, and dealing with personal responses to providing care (along with 35 corresponding tip sheets addressing each CNCC item) [4,32,34,35]. The Bakas Caregiving Outcomes Scale (BCOS) [7] with corresponding tip sheets addresses caregiver life changes, which is our primary outcome. Six skill-building tip sheets address the following: (1) strengthening existing skills, (2) screening for depressive symptoms, (3) maintaining realistic expectations and time management, (4) communicating with health care providers, (5) problem-solving, and (6) stress management [32,34,35]. The guide also includes (7) a new self-management skill-building tip sheet on goal setting designed for this study [37]. We will measure several key social determinants of health factors [48,49] at baseline, which will be useful in training caregivers on how to use skill-building strategies to address their needs and concerns within the context of their own social determinants of health. For example, 2 instrumental care tip sheets address managing finances and covering costs (financial strain is a key social determinant of health factors). The problem-solving skill-building tip sheet will help caregivers identify barriers and create new solutions to further mitigate financial strain. The goal-setting tip sheet helps caregivers set realistic goals to improve their own health (eg, setting a walking goal that considers neighborhood characteristics as another social determinant of health factor). Our original TASK, TASK II, and TASK III tip sheets have a large 14-point Arial font that is easily readable across all age groups. In our previous TASK II and TASK III studies, caregiver ages ranged from 21 to 83 years, representing a wide range of ages across the life span [34,38]. Our positive satisfaction data demonstrate that our materials are acceptable for a wide range of younger and older caregivers [38]. The TASK III resource guide is constructed to be appealing to both sexes. Male caregivers expressed high satisfaction ratings in our TASK II trial (>4.0 on a scale of 1 to 5), with positive qualitative comments (eg, “It’s been such a help to me. It gave me an outlet that I could express my feelings.”). Female caregivers also expressed high TASK II satisfaction ratings (>4.0) with positive TASK II qualitative comments (eg, “They’re not just there for the person that’s had the stroke, they’re there for the caregiver too.”). Our TASK II [34] and TASK III trials showed minority group representation consistent with the demographics of our Midwest populations, with 24.8% (63/254) in TASK II and 24% (18/74) in TASK III being Black or African American. When seeking input for the goal-setting tip sheet and technology preferences for our TASK III program, we purposefully enrolled 50% Black or African American caregivers to ensure that our ratings and qualitative data reflected their views [34,35]. In our TASK III R21 feasibility trial, we employed a diverse research team, with our project manager, one of the TASK III nurses and one of the ISR nurses who identified as Black. We believe that these efforts enhanced the success of our TASK II and TASK III trials. Although not as well represented in our area, we will welcome other minority and ethnic group individuals.

#### TASK III USB Drive

A USB drive containing a searchable PDF file of the entire TASK III resource guide and separate PDF files of each of the TASK III tip sheets will be mailed to caregivers. Instructions and links to download the TASK III e-book and access the TASK III interactive website will also be provided on the USB drive.

#### Amazon Fire Tablet

In addition to the USB drive, we will provide all stroke family caregivers enrolled in the study with an Amazon Fire Tablet to access our materials. For the TASK III group, we will load the TASK III tip sheets as PDF files and provide links for the TASK III e-book, the TASK III website, and Zoom videoconferencing.

#### TASK III e-Book

An interactive multitouch e-book for the TASK III resource guide has been designed using Kotobee software [50] and tested for feasibility for use on multiple devices (eg, desktops, laptops, phones, tablets, and e-book readers). A download link automatically installs Kotobee Reader [50] and the TASK III e-book onto any Mac or PC laptop or desktop. The TASK III e-book will also be installed on the Amazon Fire tablets given to caregivers.

#### TASK III Interactive Website

We engaged the expertise of Oohology [51] to create in WordPress a searchable TASK III PDF library with TASK III tip sheets that appear on the website based on caregiver responses to an automated assessment process containing the CNCC and BCOS. Infrastructure for backend support of these technologies was provided during our TASK III R21 feasibility study, with continued support and ongoing hosting of the website provided by Oohology [51]. Most importantly, the tip sheets can be viewed on any laptop, tablet, or smartphone. Caregivers are instructed by research staff on how to access the TASK III interactive website through a unique username and password. *All materials created for the TASK III USB drive, e-book, and website are based on our TASK III resource guide.* Embedded within the TASK III resource guide tip sheets are links to additional resources available on the internet. We will ensure that the links are active and checked regularly during project implementation. Similar to the ISR group, the TASK III group can access the American Heart Association brochure through the American Heart Association website, which offers publicly available guidelines and web-based information for family caregivers. In addition, TASK III caregivers will receive a pedometer to track their daily steps.

#### TASK III Telephone Calls

The TASK III intervention calls will take place via telephone or videoconferencing using FaceTime or Zoom per caregiver preference [38]. Similar to the TASK II intervention [34-36], caregivers will receive 8 weekly calls from a nurse, with a booster call at 12 weeks. Caregivers will be assigned to a specific nurse for consistency. Nurses will train caregivers on how to assess and prioritize their needs, concerns, and life changes, then help them select corresponding content and skill-building tip sheets. The new goal-setting tip sheet will be used during each call to promote caregiver self-management of their symptoms and health.

#### TASK III FaceTime

Caregivers may receive calls using FaceTime [52]. Over Wi-Fi, FaceTime is accessible on iPhone 4 (Apple, Inc) or later, iPad 2 (Apple, Inc) or later, or iPad Mini (all models; Apple, Inc). Android-accessible apps such as Facebook Messenger (Meta Platforms, Inc), Google Duo (Google LLC), or Skype (Skype Technologies) will be considered.

#### TASK III Zoom

Caregivers can receive calls using Zoom videoconferencing [53]. Zoom unifies cloud videoconferencing, simple web-based meetings, and a software-defined solution into 1 easy-to-use platform, offering the best video, audio, and wireless screen-sharing experience across Windows, Mac, Linux, iOS, Android, and Blackberry devices [53]. Zoom allows real-time screen-sharing of TASK III resource guide materials during calls. The university has purchased secure Zoom links for research purposes to ensure the privacy of participants, which will be used in this TASK III R01 study. The length of each TASK III session will vary based on the needs of the caregiver. As in our previous study with the TASK II program [34], our R21 feasibility trial showed that the total number of minutes on the TASK III calls averaged about 43 minutes for each of the 9 TASK III calls (mean 389, SD 198 min).

#### Differences and Overlaps Between TASK III and ISR Groups

Both the TASK III group and the ISR group will receive the American Heart Association brochure, access to the American Heart Association website, a pedometer, an Amazon Fire Tablet, a USB drive, and 8 weekly phone calls from a nurse with a booster call at 12 weeks. Only the TASK III group will receive the TASK III resource guide that contains the CNCC and BCOS to assess caregiver needs, concerns, and life changes, as well as skill-building and content tip sheets that correspond to the CNCC and BCOS items. In the TASK III group, the nurses will train caregivers on how to assess and address their own needs, concerns, and life changes using the TASK III resource guide. This guide (containing the BCOS) informs our primary hypothesis that the TASK III group, compared with the ISR group, will have greater improvements in life changes (measured by the BCOS) from baseline to 8 weeks (after the intervention). We expect that these improvements will be sustained for a longer term from baseline to 12, 24, and 52 weeks. Including the BCOS as part of the TASK III intervention will enhance the power to detect an effect on caregiver life changes as measured by the BCOS.

#### Criteria for Discontinuing or Modifying Allocated Interventions

Participation in the study is completely voluntary, and caregivers may withdraw at any time. Caregivers who ask to withdraw from the study will be given the option for a partial or full withdrawal. For example, participants may complete the rest of the calls with the nurse and withdraw from the remaining data collection interviews or participants may withdraw from the calls with the nurse and choose to complete the remaining data collection interviews. Participants may choose to completely withdraw from all study-related activities. Previously collected data from the participants will be withdrawn if specifically requested by the participant. Otherwise, collected data will be retained and analyzed, particularly for an intention-to-treat design. Participants will be withdrawn from the study if they are unable to be contacted after up to 10 attempts. A letter will be mailed to the participants notifying them that they have been withdrawn from the study.

#### Strategies to Improve Adherence to Interventions

Adherence to protocols for both ISR and TASK III groups will be a high priority for this study. The Treatment Fidelity Checklist [54] addressing design, training, delivery, receipt, and enactment will be used to maintain and track treatment fidelity for both TASK III and ISR procedures [36,54]. Training for the nurses will include detailed manuals and podcasts, training booster sessions, self-evaluation of recordings, evaluation by supervisors, quality checklists, and frequent team meetings [36]. TASK III nurses will attend an 8-hour training session to learn the intervention content and delivery processes and to role-play sessions with each other. The role-play sessions will be recorded and checked for competency before caregiver interaction. Beyond initial training, TASK III nurses will be monitored weekly during team meetings and asked to complete a self-evaluation of their recordings for adherence to the protocol on a monthly or bimonthly basis. Then, supervisors (ie, the PI or coinvestigator) will listen to recordings, analyze self-evaluations, and provide individualized feedback and retraining when necessary. ISR nurses will undergo a similar process, but the initial training session will last for 4 hours. Protocol adherence for the TASK II study was excellent at 80% (37.6/47) for the TASK II group and 92% (43.2/47) for the ISR group, and for common items on the checklist, 90% (42.3/47) in the TASK II group and 92% (43.2/47) in the ISR group [36]. Focus groups with nurses yielded further evidence for treatment fidelity [36]. The same procedures were used to track treatment fidelity in the TASK III R21 feasibility trial; this TASK III R01 study will follow the same rigorous treatment fidelity procedures with the nurses. Caregivers will be monitored for treatment fidelity regarding receipt (eg, the number of minutes spent on the calls with the nurse; ratings of the helpfulness of each call; the number of minutes of viewing TASK III or ISR materials; and the types of materials viewed, such as TASK III tip sheets or American Heart Association brochures or the website). Enactment will be monitored by asking caregivers whether their problems discussed with the nurse were unresolved, making progress, or resolved. In the TASK III group, the enactment will be further monitored by having caregivers evaluate their own progress on their goals to improve their own health based on our new TASK III goal-setting tip sheet [37].

#### Relevant Concomitant Care Permitted or Prohibited During the Trial

The trial will not interfere with any concomitant care or interventions that caregivers or their stroke survivors receive. No concomitant care or interventions are prohibited during the trial.

#### Provisions for Posttrial Care

There are no provisions for ancillary or posttrial care. This is a minimal risk study where the risk is not expected to be more than one would have in daily life.

### Outcomes

Outcomes were selected based on a conceptual model derived from our previous research on caregiver needs and concerns [4,35]; our stroke caregiver outcomes work [8-10,31,34] informed by the transactional approach to stress proposed by Lazarus [55-57]; and our conceptual model that has empirical support from our previous research [8-10,31,34], including the original TASK and TASK II programs [31-36]. We added concepts from the self-efficacy theory based on the works by Bandura [58-60] and Lorig et al [61,62] to improve caregiver health. Our outcomes are clinically relevant, particularly our primary outcome of caregiver life changes (eg, physical health, physical functioning, emotional well-being, and general health) because of providing care [7]. Caregivers are known to neglect their own health needs while providing care [4-6,35,37], making them the hidden patients in our health care system. Difficulty with tasks threatens caregivers’ ability to provide future care (ie, threat appraisal) [7-10] and is strongly associated with caregiver depressive symptoms and major life changes as a result of providing care (eg, physical health, physical functioning, emotional well-being, and general health) [7-15,17,39]. Finally, stressful caregiver experiences have been reported to impede the survivor’s rehabilitation [16-18] and to increase costs from premature long-term institutionalization [8-11,19,20]. In addition to our primary outcome of caregiver life changes, we have included several secondary outcomes, such as caregiver depressive symptoms, other symptoms, unhealthy days, self-management of diet and exercise, and self-reported health care use. Further outcomes include our mediators of caregiver task difficulty, threat appraisal (ie, ability to provide future care), and self-efficacy for maintaining a healthy diet and exercise, as well as program evaluation outcomes. As listed on the ClinicalTrials.gov website (NCT05304078), Multimedia Appendix 4 [7-10,31,32,34,48,49,63-86] details measures and time points for our primary outcome, secondary outcomes, mediators, and program evaluation outcomes.

### Participant Timeline

Table 1 provides the time schedule of enrollment, interventions, and data collection interviews for participants. Screening for eligibility and data collection interviews will take place via telephone. TASK III and ISR calls with the nurse will take place via telephone or videoconference (eg, FaceTime or Zoom) based on caregiver preference. Data collection will take place at screening; baseline; and 8-week (after the 8 weekly calls with the nurse), 12-week (after the ninth booster call with the nurse), and 24- and 52- week follow-ups. Randomization will occur after the completion of the baseline interview.

**Table 1:** Schedule of the study period, including enrollment, interventions, and assessments.

Time point			Enrollment		Allocation		Postallocation period											Close out	
			Screen		Baseline		8 weekly nurse calls		8 wk		9th nurse call		12 wk		24 wk		52 wk		
**Enrollment**																			
	Eligibility screen	✓																	
	Informed consent	✓		✓															
	Allocation			✓															
**Intervention**																			
	TASK III^a^ group					✓				✓									
	ISR^b^ group					✓				✓									
**Assessments**																			
	Demographics, comorbidities, and other social determinants of health			✓															
	Survivor impairment			✓				✓				✓		✓		✓			
	Primary, secondary, and other outcomes (mediators)			✓				✓				✓		✓		v			
	Program evaluation outcomes											✓							

^a^TASK III: Telehealth Assessment and Skill-Building Kit.

^b^ISR: information, support, and referral.

Primary, secondary, and other outcomes (ie, mediators and program outcomes) are detailed in the *Outcomes* section. Further information about these measures, as well as demographics, comorbidities, social determinants of health, and survivor impairment, is provided in Multimedia Appendix 4.

### Sample Size

Sample size is based on the change in the total BCOS [7] scores from baseline to 8 weeks for the primary outcome of caregiver life changes because of providing care (ie, physical health, physical functioning, emotional well-being, and general health). For caregiver life changes (BCOS) [7], the sample size of 132 in each group will provide 80% power to detect a mean difference of 0.9 in the change from baseline, with the TASK III group showing a mean difference of 4.0 and ISR group showing a mean difference of 3.1 and an estimated SD of 2.6 [34]. We will also have sufficient power to address secondary outcomes. A sample size of 132 in each group will achieve 80% power to detect a mean difference of 0.20 in the change from baseline in depressive symptoms at 8 weeks between groups, with a mean difference of –0.80 in the TASK III group and –0.60 in the ISR group and an estimated SD of 0.70 in both groups using a 2-sample *t* test with a significance level of 0.05 [34]. This detectable difference is lower than the difference in depressive symptoms observed in the pilot TASK III R21 study. On the basis of pilot data, it is estimated that there will be 124 (42%) caregivers who screen positive for mild to severe depressive symptoms at baseline (ie, Patient Health Questionnaire-9 [PHQ-9] Depression Scale score >5). Because the randomization will be stratified by baseline depressive symptoms (see the *Sequence Generation* section), we estimate 62 caregivers with mild to severe depression in each group. A sample size of 62 in each group will achieve 80% power to detect a mean difference of 0.60 in the change from baseline in depression symptoms at 8 weeks between groups, with a mean difference of –2.60 in the TASK III group and −2.00 in the ISR group and an estimated SD of 1.20 in both groups, using a 2-sample *t* test with a significance level of 0.05 [34]. This detectable difference is lower than the difference in depression symptoms observed in the pilot TASK III R21 study. For unhealthy physical and mental days, the sample size will provide 80% power to detect a rate ratio of 0.85 (a 15% reduction in unhealthy days) [34]. The planned enrollment is 296 (n=148, 50% in the TASK III group and n=148, 50% in the ISR group) to account for 10.8% (16/148) attrition [34]. Power Analysis & Sample Size (version 12; NCSS LLC) was used to produce all power estimates. Power calculations were not performed for the additional secondary outcomes (ie, other symptoms, self-management steps, exercise self-management, diet self-management, and health care use).

### Recruitment

**Figure 2:**
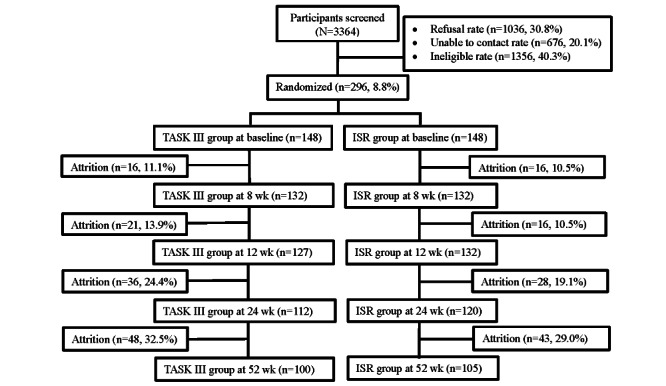
Proposed participant flow diagram. The values are provided on the basis of the Telehealth Assessment and Skill-Building Kit (TASK III) R21 (R21NR016992; ClinicalTrials.gov ID: NCT03635151) feasibility study, except for the 24-week and 52-week values, which were based on the Telephone Assessment and Skill-Building Kit (TASK) II R01 (R01NR010388; ClinicalTrials.gov ID: NCT01275495) study. ISR: information, support, and referral.

Recruitment methods and retention rates were successful in our previous studies [31,34] and even better in our most recent TASK III R21 feasibility study. On the basis of our recruitment success with our TASK II and TASK III R21 feasibility studies, we plan to screen 3364 caregivers and randomly assign at least 296 caregivers to achieve our goal of 148 (50%) caregivers per group (Figure 2). Accounting for 10.8% (16/148) attrition, this should allow us to have at least 132 (89%) caregivers per group at our primary 8-week time point.

Screening 3364 caregivers over 3 years (1121 per year) is feasible given our strong track record of collaboration and the large number of stroke survivors treated yearly at each recruitment site. As in our previous studies, bioinformaticians, stroke coordinators, or other employees from each recruitment site will provide us with lists of stroke survivors and their next of kin. As approved by our university IRB, study flyers signed by the stroke survivor’s physician, nurse, director of the stroke team, along with copies of the informed consent form, will be mailed to caregivers of survivors receiving care from each recruitment site. A telephone number is provided for stroke caregivers to call if they do not wish to be contacted for the study. Approximately 1 week after the study flyers and informed consent forms are mailed, caregivers will be contacted via telephone by research staff to determine their interest and screen for eligibility. Flyers with response cards may also be distributed by staff employed at each recruitment site.

### Assignment of Interventions: Allocation

#### Sequence Generation

Caregivers will be randomly assigned 1:1 to the TASK III or ISR group using a permuted block randomization stratified by type of relationship (spouse vs adult child or other) and baseline depressive symptoms (nondepressed vs depressed, with a PHQ-9 score ≥5) as in the TASK II trial [34]. Random permutations within each block within each stratum will be generated using a random number generator (SAS Proc Plan; SAS Institute).

#### Concealment Mechanism

The randomization scheme will be concealed in an Excel (Microsoft Corporation) document uploaded to the REDCap project site that will be accessible only to the biostatistician. Randomization will occur by logging into the REDCap project website and entering the type of relationship (spouse vs adult child or other) and baseline depressive symptoms (nondepressed vs depressed, with a PHQ-9 score ≥5) for group assignment (TASK III vs ISR).

#### Implementation

The biostatistician will generate the allocation sequence and upload it as an Excel file to the REDCap project site as a concealed file. The biostatistician will then program the REDCap project site to generate the group assignment once the PI or the project manager enters the type of relationship and baseline depressive symptoms. After baseline data collection, data collectors will notify the PI or the project manager that the caregiver is ready for randomization. Data collectors will not be involved in the randomization process.

### Assignment of Interventions: Blinding

#### Who Will Be Blinded

Data collectors will be blinded to group assignment to mitigate potential bias during the 8-, 12-, 24-, and 52-week follow-up interviews. Blinding will be maintained by restricting data collector access to group assignments in REDCap. Separate weekly research team meetings will be conducted with the data collectors. Caregiver participants will be instructed not to tell the data collectors which group they have been assigned to. Caregiver trial participants, the nurses, the project manager, the PI, and a coinvestigator will be unblinded so that the proper TASK III or ISR materials can be mailed to the participants and the correct TASK III or ISR protocol can be followed. The PI and the coinvestigator will closely monitor treatment fidelity for the TASK III and ISR groups. Separate nurses will be used for the TASK III or ISR groups to avoid treatment diffusion. We have a strong track record of keeping TASK III and ISR groups separate, including strict monitoring of treatment fidelity and adherence to protocol [36]. The lead biostatistician and her master’s degree–prepared staff statistician will be unblinded to create reports, assess for missing data, and explore data trends in preparation for Data Safety and Monitoring Plan reports. While analyzing data trends for Data Safety and Monitoring Plan reports, the PI and the research team will be blinded as to which group represents each outcome variable within data tables by labeling each group by a letter rather than using the TASK III or ISR abbreviations.

#### Procedure for Unblinding if Needed

Only data collectors will be blinded during the trial. Unblinding the data collectors will not be permissible.

### Data Collection and Management

#### Plans for Assessment and Collection of Outcomes

Multimedia Appendix 4 lists the study measures. Most measures were used in our previous studies [7-10,31,32,34]. All measures demonstrated acceptable evidence of reliability and validity, except the Caregiver Evaluation of Technology Scale, which was created specifically for this study. We will assess the reliability and validity of this scale, and if unacceptable, we will analyze it at the item level. Using the measures in Multimedia Appendix 4, the estimated time for telephone interviews for each time point is 60 minutes for baseline, 33 minutes for 8 weeks, 39 minutes for 12 weeks, and 33 minutes for 24 and 52 weeks. Telephone interviews of this length have not been found to be burdensome in our previous studies [7-10,31,32,34].

#### Plans to Promote Participant Retention and Complete Follow-Up

To promote participant retention and complete follow-up, data collection interviews and TASK III or ISR nurse calls will be made to caregivers at times that are convenient to them. Once a baseline interview is scheduled, REDCap will prepopulate a schedule for all remaining data collection interviews and nurse calls, usually on the same day of the week and time of the day. Using the calendar function in REDCap, the schedule will appear on the calendar while maintaining blinding of data collectors to group assignments. The schedule will be mailed to each caregiver, with assurance that days and times can be rescheduled as needed. A detailed calling protocol will be used by data collectors and nurses, along with call tracking entered within REDCap. To further promote retention and follow-up, caregivers who ask to withdraw from the study will be offered the option to participate in a final data collection interview that contains all the primary, secondary, mediators, and program evaluation outcomes, as specified for the 12-week time point as listed in Multimedia Appendix 4. Consistent with our intention-to-treat design, caregivers who do not complete all 8 weekly calls with the nurse or the ninth booster call with the nurse will still be asked to continue in the trial for the remaining data collection calls.

#### Data Management

Data management will be overseen by the PI, coinvestigator, project manager, and the lead biostatistician and staff statistician. All data collection interviews and TASK III and ISR nurse calls will be audio recorded for quality assurance. During monthly or bimonthly self-evaluations with nurses and data collectors, audio recordings will be checked for adherence to the protocol and accuracy in data entry. All study data and tracking will be entered into an electronic data system, REDCap [87], which is a secure research electronic data management system with validated data entry, audit trails for tracking data manipulation, and export procedures. It is HIPAA compliant, satisfying all local, state, and federal regulations for the capture and storage of private health information for research purposes. The biostatistician will run quarterly reports assessing for missing data and range checks for data values. Any missing data or suspected errors in data entry will be discussed with the PI and the project manager, who can then access the audio recordings to resolve any issues with data entry.

### Statistical Methods

#### Recruitment, Attrition, and Fidelity Ratings of Data Collection and Intervention Procedures

Using procedures similar to the TASK II study and our TASK III feasibility study, we will monitor screening and recruitment rates, attrition rates, and fidelity ratings of all data collection and intervention procedures [34,36,54,88]. We will compute the number of participants screened and enrolled per month, the proportion of screened eligible participants who enroll, intervention assignment–specific retention rates at each follow-up visit, and the proportion of outcome measures completed. For fidelity ratings, we will use an itemized checklist to monitor adherence to the unique components of the TASK III study [36]. Adherence will be scored with dichotomous responses for the presence or absence of each item. Frequencies and percentages will be calculated for each item by group. Intervention dosage will be calculated for nurse call duration (min) and the time caregivers spend reading study materials (min) for each group. Descriptive statistics for intervention dosage will be computed by group, and between-group mean differences will be evaluated using 2-sample *t* tests or nonparametric alternatives if the normality assumption is violated.

#### Assessment of Scales and Descriptive Statistics

After initial data verification, cleaning, and scale scoring, the coefficient alpha will be calculated as a measure of internal consistency reliability on all multiple-item scales. Descriptive statistics (frequencies, measures of central tendency, and variability) will be produced for all variables. Data will be screened for outliers, multicollinearity, and statistical model assumptions.

#### Baseline Equivalence and Possible Covariates

Baseline differences in caregiver and survivor characteristics and social determinants of health will be explored to identify potential covariates for our analyses. As in our TASK II trial, stratified random assignment using the type of relationship and baseline depressive symptoms will ensure a balance between the 2 groups for those 2 factors [34]. Baseline differences between the 2 groups will be evaluated using 2-sample *t* tests or Wilcoxon rank sum tests, chi-square tests, or Fisher exact test as appropriate. Baseline variables that are significantly different between the 2 groups at the .05 level will be controlled for in all analyses. In our previous TASK II trial, the ISR calls, on average, were significantly shorter than TASK II calls (*P*<.01) [34]. As in our previous studies [31,34], the number of minutes on TASK III and ISR calls with the nurse may serve as a covariate in our analyses.

### Primary Analyses for Specific Aims 1 and 2

#### Overview

The general modeling strategy for the analyses in aims 1 and 2 will be repeated measures regression modeling using the generalized liner mixed model (GLMM) framework. In GLMM, it is possible to use data from participants when some of the data are missing under the assumption of missing at random. In addition, this modeling strategy allows for time-varying covariates, flexible covariance structures, and participant-specific effects and thus will be able to accommodate the analyses proposed. Our primary goal will be to assess the overall intervention effects on the change from baseline. All primary analyses conducted will be based on the intention-to-treat principle. Analyses will be done using SAS software (version 9.4 or higher).

#### Specific Aim 1

The *primary outcome* is caregiver life changes as a result of providing care (ie, physical health, physical functioning, emotional well-being, and general health). Life changes are measured using the BCOS total score [7]. The explanatory variables used in the specific aim 1 model will be the intervention group, time, and the intervention group–by–time interaction. Covariates (including social determinants of health) identified using the process described above will be included as appropriate. The number of minutes spent on calls with the nurse in both TASK III and ISR groups will potentially be used as a covariate as well. The model examined will be a 2 (intervention: ISR and TASK III) by 5 (time: baseline and 8, 12, 24, and 52 wk) mixed factorial model, along with covariates. In the GLMM framework, an appropriate link function will be specified depending on the distribution of the dependent variable. Hypotheses related to the immediate postintervention time point at 8 weeks and long-term, sustained efficacy at 12, 24, and 52 weeks will be tested using relevant least square mean contrasts and simple effects constructed within SAS PROC GLIMMIX.

#### Specific Aim 2

S*econdary caregiver outcomes* are depressive symptoms; other symptoms (stress, fatigue, sleep, pain, and shortness of breath); unhealthy days; self-management of diet and exercise; and self-reported health care use. In the analyses for depressive symptoms, we will look at both overall and subgroup analyses for caregivers who screen positive for depressive symptoms (PHQ-9 score >5) [34]. *Mediators* are task difficulty, threat appraisal, and self-efficacy for exercise and diet. Each of these outcomes will be modeled separately. The models for secondary caregiver outcomes and mediators used in specific aim 2 are similar to those for life changes tested for aim 1 and will include the intervention group, time, and the intervention group–by–time interaction. Covariates (including social determinants of health) identified using the process described above will be included as appropriate, as well as potentially the number of minutes spent on the calls with the nurse in both TASK III and ISR groups. The full model consists of a 2 (intervention: ISR and TASK III) by 5 (time: baseline and 8, 12, 24, and 52 wk) mixed factorial, along with covariates. In the GLMM framework, an appropriate link function will be specified depending on the distribution of the dependent variable. Hypotheses related to immediate postintervention time point at 8 weeks and long-term, sustained efficacy at 12, 24, and 52 weeks will be tested using relevant least squares mean contrasts and simple effects constructed within SAS PROC GLIMMIX. *Program evaluation outcomes* consist of caregiver satisfaction and evaluation of technology ratings. Satisfaction ratings from the Caregiver Satisfaction Scale [32] (usability, ease of use, and acceptability) and evaluation of technology ratings will be summarized at the item and scale levels by intervention group using descriptive statistics, including mean and 95% CI. Caregiver Satisfaction Scale scores, total, and subscales will be compared between groups using 2-sample *t* tests or nonparametric alternatives if the normality assumption appears violated.

#### Interim Analyses

There are no plans for interim efficacy or futility analysis. There is little chance that we would find harm from the intervention, thus rendering interim efficacy and futility analysis inappropriate.

### Methods for Additional Analyses, Including Subgroup Analyses

#### Age Across the Life Span

We will compare caregiver age between TASK III and ISR groups using 2-sample *t* tests and control for age as a covariate in our analyses if significant age differences are noted.

#### Sex as a Biological Factor

We will explore sex differences by careful description of subgroup effects.

#### Minority Group Representation

We will also explore racial differences (ie, Black or African American vs White individuals) by careful description of subgroup effects.

### Methods to Handle Protocol Nonadherence and Statistical Methods to Handle Missing Data

We will make every effort to avoid missing data. If missing data are identified, the PI or the project manager will access the corresponding audio recording to determine whether missing data were due to an error in data entry. We will assess the reasons, patterns, and distribution of missing data, allowing us to assess whether an assumption of missing completely at random is reasonable or whether missingness is conditional on another variable in the dataset (ie, missing at random). Descriptive statistics will compare the characteristics of patients with and without missing data. If missing at random, we will incorporate variables that are identified to be related to the missingness in the analysis using multiple imputations if the amount of missing data affects the study results. Missing data have been minimal for our original TASK study [31], TASK II randomized controlled clinical trial [34], and TASK III feasibility study; we will use the same procedures to avoid missing data in this study. As mentioned previously, we will use an intention-to-treat design and rigorous treatment fidelity procedures [36] to monitor all research staff for adherence to protocol.

### Plans to Give Access to the Full Protocol, Participant-Level Data, and Statistical Code

We will make the full protocol, participant-level data, and statistical code available to others. Data will be shared with undergraduate honors students, graduate students, postdoctoral students, and interested faculty members at our university and other institutions. Special measures will be taken to ensure that family caregivers and stroke survivors are not identified by any data that are shared. Close collaboration with the PI and her research team will be necessary for the use of shared data. Project-generated resources will include our finalized REDCap database, intervention materials, and training manuals for the project manager, TASK III study nurses, ISR study nurses, data collectors, and screening and recruitment staff. Close collaboration with the PI and her research team will be necessary for the use of shared resources by other investigators.

### Oversight and Monitoring

#### General Composition of the Research and Intervention Delivery Team

##### Overview

The study team will consist of TB (PI and nurse), who will lead the interdisciplinary team of coinvestigators, consultants, TASK III and ISR nurse interveners, project coordinators, and data collectors to successfully achieve the aims of the study. EM (coinvestigator and nurse) will help to guide and train the TASK III and ISR nurses, especially with respect to tracking and maintaining treatment fidelity throughout the intervention period. NK (coinvestigator and physician) will provide valuable expertise on stroke, facilitate recruitment of participants, and provide guidance on future implementation of TASK III intervention into stroke systems of care. BH (coinvestigator and biomedical informatics specialist) will facilitate accessing Epic medical records to obtain lists of stroke survivors and their next of kin for recruitment. HS (coinvestigator and biostatistician) will work with investigators and a staff statistician to develop and implement the statistical analysis plan and dissemination of results. MR (coinvestigator and IT and instructional designer specialist) will oversee the TASK III telehealth technologies and work with an information technologist and website or digital designer for ongoing support and hosting of the TASK III website. KD (coinvestigator and physical therapist) will help promote physical activity in caregivers, provide oversight and interpretation, and assist with the dissemination of physical activity–related data. BB (consultant and dietician) will provide ongoing expertise for study methods and materials related to the caregivers’ dietary habits, as well as oversight of the interpretation and dissemination of nutrition-related data. MM (consultant and social worker) will consult on updating and maintaining equitable and accessible community resources and support for both TASK III and ISR. HJ (consultant and nurse) will provide consultation on culturally sensitive approaches for recruiting and interacting with diverse family caregivers. JKA and PHM (consultants and nurses) will be available for consultation as needed for the overall study design and methods.

In addition to the PI, coinvestigators, and consultants, the team will consist of the following staff.

##### Project Manager

The person selected for this role has a master’s degree in public health and will coordinate all aspects of the study including all day-to-day project operations, first-line supervision of research staff, coordination of communication with all study personnel, research team meetings, preparation of agendas and minutes, assistance in the preparation of the training manuals and protocols, maintenance of study files, budget management, scheduling and coordination of all family caregiver calls and mailings, and study coordination with recruitment sites. Furthermore, they will conduct caregiver and stroke survivor screenings and informed consent procedures. Under the direction of the biostatistician and the PI, they will also assist in the development, testing, and maintenance of the REDCap database; conduct the query management process; provide data management documentation, such as the data management plan; and ensure that data are being entered and cleaned in a timely and appropriate manner.

##### Nurse Interveners

A total of 3 hourly nurse interveners will be hired and trained to deliver the 9 TASK III intervention calls to 148 stroke caregivers randomly assigned to the TASK III intervention group. Three additional hourly nurse interveners will be hired and trained to deliver the 9 ISR group calls to 148 stroke caregivers randomly assigned to the ISR group. Nurses will be required to have a current registered nurse license and excellent interpersonal skills. In addition to making caregiver calls, the nurses will be responsible for self-evaluation of their own audiotapes, further retraining procedures, and documentation of TASK III intervention or ISR content delivered to caregivers. They will also attend weekly research team meetings.

##### Research Assistants

A total of 4 hourly research assistants will be hired and trained to conduct participant screening and collect data via telephone for 296 stroke caregivers at the 5 time points specified for the project (baseline and 8, 12, 24, and 52 wk). The hourly research assistants will provide coverage for each other in the event of illness or time off, cover a wide range of availability times for caregiver data collection calls, and attend weekly research team meetings while remaining blinded to group assignment of caregivers throughout the study.

##### Informatics Support Person

The informatics support person for this study is a data analyst from the Center for Health Informatics at the University of Cincinnati Department of Biomedical Informatics. The data analyst will be supervised by BH to extract lists of stroke survivors and their next of kin from Epic electronic medical records from the University of Cincinnati Health and University of Cincinnati Health West Chester Hospitals for recruitment.

##### IT Support and Website or Digital Designer

This person will be supervised by MR and will work closely with TB and her interdisciplinary team to refine and maintain the TASK III telehealth technologies for the TASK III resource guide (ie, mailed hard copy, USB drive, Amazon Fire Tablets, e-book, and TASK III website) and the technologies for nurse calls (ie, telephone, Zoom videoconferencing, and FaceTime on iOS devices). The IT support and website or digital designer will work with Kotobee [50] on the TASK III e-book and Oohology [51] for ongoing support and hosting of the TASK III website. He will also help to train TASK III and ISR nurses on the use of the TASK III telehealth technologies and provide ongoing IT support throughout the project.

#### Staff Biostatistician

The staff biostatistician will work with HS to carry out the statistical analysis plan to address the study aims.

#### Composition of the Data Monitoring Committee, Its Role, and Reporting Structure

This study was approved by the university IRB as “no greater than minimal risk.” According to the funder’s policy (ie, National Institute of Nursing Research; NINR), “Monitoring by the PI or designee may be appropriate for protocols involving minimal risk or no more than a minor increase over minimal risk which are conducted at a single site.” For this study, the IRB- and NINR-approved data and safety monitoring plan will be the responsibility of TB (the PI). Members of the research team will provide input, particularly HS, the statistician coinvestigator; EM, the nurse coinvestigator; and NK, the physician coinvestigator. The PI (TB) and research team members (HS, EM, and NK) are well-qualified and will meet quarterly (and more frequently as needed) to review data and the safety of the study procedures and involvement of participants. TB (the PI) will consult with the research team members (HS, EM, and NK), but she will be the monitoring entity for this study. She will collaborate closely with our local IRB throughout the study and will submit the approved IRB-continuing review, including the dates and summarized reports generated from data and safety monitoring meetings with the research team to the National Institutes of Health (NIH) along with the yearly NIH progress report. There are no conflicts of interest with or financial stakes in the research outcome with the PI (TB) or any members of the research team.

#### Adverse Event Reporting and Harms

The PI (TB) will be seeking input from members of the research team (HS, EM, and NK), and will meet quarterly (and more frequently as needed) with them to (1) review any deviations in the study protocol; (2) review any adverse events; (3) track any negative trends in data that may indicate harmful effects of the study procedures; (4) review study recruitment and retention; and (5) review data management procedures, particularly those designed to protect the privacy of participants. All protocol deviations, adverse events, and unanticipated problems will be reported by the PI (TB) to the local IRB and NINR yearly or more frequently as needed. At the time of the yearly IRB-continuing review, a summary of the frequency and dates of the data and safety monitoring meetings with the PI (TB) and the research team (HS, EM, and NK) will be provided, along with reviews and reports generated from each meeting. The approved IRB-continuing review, including the dates and summarized reports generated from the data and safety monitoring meetings with the PI and the research team, will be submitted to the NINR along with the yearly NINR progress report. The PI (TB) will provide a report of the following to IRB and NINR within 48 hours: (1) unanticipated problems or unexpected serious adverse events that may be related to study protocol; (2) IRB-approved revisions to the study protocol that indicate a change in risk for participants; (3) a summary of recommendations made by the PI and the research team as appropriate and, if applicable, the action plan for response; and (4) notice of any actions taken by the IRB or regulatory bodies regarding the research and any responses to those actions. All personal identifiers will be removed from any documents sent to the NINR. Identifiable data will be stored in locked file cabinets and within our secure, password-protected, HIPAA-compliant REDCap database.

#### Frequency and Plans for Auditing Trial Conduct

The PI (TB), with inputs from the research team (HS, EM, and NK), will provide auditing of at least 50% of all cases for compliance with IRB requirements, conformance with informed consent requirements, verification of source documents, and investigator compliance. Recommendations concerning the continuation or conclusion of the trial will be made during each quarterly meeting, based on the results of monitoring. The process will involve both the investigators and the sponsor (NINR).

#### Plans for Communicating Important Protocol Amendments to Relevant Parties

The PI will communicate important protocol modifications with the coinvestigators, consultants, project manager, and research staff as appropriate. All modifications will be approved by the local IRB before implementation and reported to the NINR on the yearly NIH progress report.

### Dissemination Plans

This clinical trial is registered (ClinicalTrials.gov ID: NCT05304078), and information about the results will be submitted to ClinicalTrials.gov, which is available to the public. The IRB-approved informed consent form will include the following statement: “A description of this clinical trial will be available on ClinicalTrials.gov, as required by the US Law. This website will not include information that can identify you. At most, the website will include a summary of the results. You can search this website at any time.” Participants will be encouraged to check back with the ClinicalTrials.gov site for a lay summary of the results after completion of the study. Participants contacting the PI for results will be referred to the ClinicalTrials.gov website. We anticipate that there will be manuscripts, not only from the findings addressing specific aims 1 and 2 but also from secondary analyses of data generated from this study. We will disseminate results from specific aims 1 and 2, as well as results from secondary analyses, in peer-reviewed journals. We will make the data available to others as detailed in the Plans to Provide Access to the *Full Protocol, Participant-Level Data, and Statistical Code* section.

## Results

Enrollment and random assignment of the first participant was on November 30, 2022, with an anticipated completion of recruitment by November 30, 2025. The primary end point data analysis is anticipated to be completed by August 31, 2026, with reporting of results in ClinicalTrials.gov anticipated to be by April 1, 2027. As of October 9, 2024, a total of 198 (66.9% of the proposed total sample of N=296) family caregivers have been enrolled and randomly assigned to the TASK III group (n=98, 49.5%) or the ISR group (n=100, 50.5%). On the basis of reviewer comments during the funding process, the exclusion criteria were changed from caregivers or survivors aged <21 years to those aged <18 years. An IRB modification was added to remove the word “unpaid” from the inclusion criteria for family caregivers because some states provide financial resources to family caregivers. Additional IRB-approved recruitment sites have been added, including the use of social media (eg, Facebook; Meta Platforms, Inc) for recruitment [89]. The protocol reported in this paper is protocol version 5, as approved by the IRB on May 9, 2024.

## Discussion

### Anticipated Results and Interpretation

This study protocol builds upon our findings from our previous studies with both the TASK [31,32] and TASK II R01 programs [34-36], as well as our most recent refinement and feasibility testing of the TASK III R21 program [37,38]. In caregivers with mild to severe depressive symptoms, the TASK II (in comparison with ISR) intervention was found to reduce depressive symptoms up to a year after baseline; however, improvement in caregiver life changes (ie, physical health, physical functioning, emotional well-being, and general health) occurred only from baseline to 12 weeks in this subsample [34]. Furthermore, TASK II caregivers typically waited until nurse calls 5 through 9 to address their own physical and emotional health needs [36]. To strengthen our findings regarding caregiver life changes, we refined the TASK III intervention to include an assessment of life changes using the BCOS [7], starting with nurse call 2, and added a goal-setting tip sheet to improve caregiver health [37]. Promising data trends indicating stronger caregiver life changes in the TASK III R21 intervention compared to the ISR intervention were reported on the ClinicalTrials.gov website. In this proposed trial, relative to the ISR group, we anticipate that caregivers randomly assigned to the TASK III group will show greater improvements in our primary outcome of caregiver life changes from baseline to 8 weeks (immediately after the intervention) in the total sample, which is sustained for a long term from baseline to 12, 24, and 52 weeks. This study will enable us to (1) successfully conduct a large efficacy trial of the TASK III intervention and (2) further refine the TASK III intervention via program evaluation data (satisfaction and technology ratings) for future implementation. Findings will provide evidence of efficacy for the TASK III program, which is a necessary step toward implementing the TASK III program into stroke systems of care.

### Potential Pitfalls, Alternative Approaches, and Future Directions

We have a strong track record of recruitment and retention in our previous TASK, TASK II, and TASK III studies [31,34]. Nevertheless, we will closely monitor recruitment and retention and will secure additional recruitment sites if needed. Our study does not require face-to-face interactions with caregivers; therefore, we are not limited by geographic boundaries for recruitment. Some caregivers might be reluctant to try the new technologically enhanced TASK III resource guide (TASK III USB drive, e-book, and interactive TASK III website) or use videoconferencing (FaceTime and Zoom) for calls with the nurse. During the first TASK III session, we will instruct and demonstrate how to use the new Amazon Fire Tablet; how to connect with the nurse via FaceTime (if they have an iOS device); how to connect with the nurse via Zoom; and how to use each TASK III resource guide delivery mode (TASK III USB drive, e-book, and interactive TASK III website) and the American Heart Association website. We will encourage caregivers to explore these newer technologies during each call from the nurse but respect their preferences [38] and suggest that they call us if they have difficulties. We will have IT support available for caregivers. For the ISR group, during the first call, we will instruct and demonstrate how to use the new Amazon Fire Tablet, how to connect with the nurse via FaceTime (if they have an iOS device), how to connect with the nurse via Zoom, and how to access the American Heart Association website.

Our future directions include procedures detailed in the *Dissemination Plans* section that include reporting our findings in ClinicalTrials.gov (ID NCT05304078) and peer-reviewed journals and making our data available to others. Once we are able to demonstrate that TASK III intervention is efficacious, our next goal is to translate the optimized TASK III intervention into stroke systems of care to meet recommendations and guidelines for follow-up care for survivors and caregivers.

## Appendix

Multimedia Appendix 1 List of recruitment sites.

Multimedia Appendix 2 The study informed consent statement.

Multimedia Appendix 3 Screenshots of the Telehealth Assessment and Skill-Building Kit III website.

Multimedia Appendix 4 Measures for primary and secondary outcomes, mediators, program evaluation outcomes, and sample characteristics.

Multimedia Appendix 5 Peer review report from the CMGC - Clinical Management in General Care Settings Study Section (National Institutes of Health, USA).

## Abbreviations

BCOS: Bakas Caregiving Outcomes Scale
CNCC: Caregiver Needs and Concerns Checklist
GLMM: generalized liner mixed model
HIPAA: Health Insurance Portability and Accountability Act
IRB: institutional review board
ISR: information, support, and referral
NIH: National Institutes of Health
NINR: National Institute of Nursing Research
PHQ-9: Patient Health Questionnaire-9
PI: principal investigator
R01: R01NR010388; ClinicalTrials.gov ID: NCT01275495
R21: R21NR016992; ClinicalTrials.gov ID: NCT03635151
REDCap: Research Electronic Data Capture
TASK III: Telehealth Assessment and Skill-Building Kit
TASK: Telephone Assessment and Skill-Building Kit

## Data Availability

The datasets generated or analyzed during this study are available from the corresponding author on reasonable request. The SPIRIT (Standard Protocol Items: Recommendations for Interventional Trials) checklist based on the SPIRIT guidelines [89-91] and the CONSORT-EHEALTH (Consolidated Standards of Reporting Trials of Electronic and Mobile Health Applications and Online Telehealth) checklist [92] based on the CONSORT (Consolidated Standards of Reporting Trials) guidelines [88] are available upon request.
