# Supplementary material for: Examining the Efficacy of the Telehealth Assessment and Skill-Building Kit (TASK III) Intervention for Stroke Caregivers: Protocol for a Randomized Controlled Clinical Trial
Source: JMIR Res Protoc. 2025 Mar 25;14:e67219. doi: 10.2196/67219 (PMC11979539; doi:10.2196/67219)
Supplement: Multimedia Appendix 1 [file resprot_v14i1e67219_app1.pdf]

List of Recruitment Sites as of October 9, 2024

- University of Cincinnati Medical Center (UC Health), Cincinnati, OH, USA
- UC Health West Chester Hospital, West Chester, OH, USA
- Mercy Health - Fairfield Hospital, Fairfield, OH, USA
- Mercy Health - West Hospital, Cincinnati, OH, USA
- Mercy Health - Anderson Hospital, Cincinnati, OH, USA
- Mercy Health - Clermont Hospital, Batavia, OH, USA
- Mercy Health - Kings Mills Hospital, Mason, OH, USA
- The Jewish Hospital – Mercy Health, Cincinnati, OH, USA
- TriHealth - Bethesda North Hospital, Cincinnati, OH, USA
- TriHealth - Good Samaritan Hospital, Cincinnati, OH, USA
- The Christ Hospital Health Network, Cincinnati, OH, USA
- Indiana University Health (IU Health) Methodist Hospital, Indianapolis, IN, USA
- Social Media - Facebook
