# Supplementary material for: Examining the Efficacy of the Telehealth Assessment and Skill-Building Kit (TASK III) Intervention for Stroke Caregivers: Protocol for a Randomized Controlled Clinical Trial
Source: JMIR Res Protoc. 2025 Mar 25;14:e67219. doi: 10.2196/67219 (PMC11979539; doi:10.2196/67219)
Supplement: Multimedia Appendix 2 [file resprot_v14i1e67219_app2.pdf]

University of CINCINNATI

UNIVERSITY OF CINCINNATI SOCIAL, BEHAVIORAL, AND EDUCATIONAL CONSENT TO PARTICIPATE IN A RESEARCH STUDY

**STUDY TITLE:**<br><br>Telehealth Assessment and Skill-Building Intervention for Stroke Caregivers (TASK III)	
**PRINCIPAL INVESTIGATOR NAME:**<br><br>Tamilyn Bakas, PhD, RN, FAHA, FAAN	**PHONE NUMBER (24-hour Emergency Contact)**<br><br>(513) 558-2254 or Tamilyn.bakas@uc.edu
**FACULTY ADVISOR (if PI is student):**<br><br>Not Applicable.	**DEPARTMENT:**<br><br>College of Nursing

KEY INFORMATION

**Purpose of the Study:**	The purpose of this study is to test a program for family caregivers of stroke survivors. The program is called the Telehealth Assessment and Skill-Building Kit (TASK III). We are comparing the TASK III program with another program called the Information Support and Referral (ISR) program.<br><br>Taking part in this study is completely up to you. It is completely voluntary, and you can withdraw or stop at any time. Your care or your family member's care will not be affected at all by your decision.
**Length of the Study:**	Family caregivers will receive 8 weekly calls from a nurse, with final call a month later. In addition, you will be asked to take part in 5 data collection interviews: one at the beginning of the program, one at 8 weeks, one at 12 weeks at the end of the program, and at 6 months and 1 year. You can choose to have these calls and data collection interviews by telephone or by videoconference. In total, there will be 9 calls from a nurse and 5 data collection interviews over the course of one year.
**Risks:**	Some questions may make you feel uncomfortable. You can refuse to answer any questions that you don't want to answer. The risk is not expected to be more than you would have in daily life.
**Benefits of the Study:**	You will probably not get any benefit from taking part in this study. But, being in this study may help caregivers better understand their needs.
**Alternative procedures:**	If you do not want to take part in this research study you may simply not participate. Your participation in this study is completely voluntary. You may withdraw at any time.

University of CINCINNATI

INTRODUCTION

You are being asked to take part in a research study. Please read this paper carefully and ask questions about anything that you do not understand. This research is funded by the National Institute of Nursing Research (NINR).

WHO IS DOING THIS RESEARCH STUDY?

The person in charge of this research study is Dr. Tamilyn Bakas of the University of Cincinnati (UC) College of Nursing. There may be other people on the research team helping at different times during the study.

WHAT IS THE PURPOSE OF THIS RESEARCH STUDY?

The purpose of this study is to test a program for family caregivers of stroke survivors. The program is called the Telehealth Assessment and Skill-Building Kit (TASK III). We are comparing the TASK III program with another program called the Information Support and Referral (ISR) program.

WHO WILL BE IN THIS RESEARCH STUDY?

About 300 family caregivers of stroke survivors will take part in this study. Family caregivers are family members or close friends caring for a person with stroke.
You may be in this study if you are:

- A family member or significant other providing care for a stroke survivor at home
- Fluent in the English language (i.e., able to read, speak, and understand English)
- Have access to a telephone or computer.
- Have no difficulties hearing or talking by telephone or computer.
- Score 4 or more on a 6-item cognitive impairment screener.
- Willing to participate in 9 calls from a nurse and 5 data collection interviews

You may not be in this study if the survivor:

- Had not had a stroke.
- Did not need help from a caregiver.
- Resides in a nursing home or long-term care facility.

You may not be in this study if the caregiver or survivor is:

- Under the age of 18 years.
- A prisoner or on house arrest.
- Pregnant.
- Has a terminal illness (e.g., late stage cancer, end-of-life condition, renal failure requiring dialysis).
- Has a history of Alzheimer's, dementia, or severe mental illness (e.g., suicidal tendencies, schizophrenia, severe untreated depression or manic-depressive disorder).
- Has a history of hospitalization for alcohol or drug abuse within the past 5 years.

University of CINCINNATI

WHAT WILL YOU BE ASKED TO DO IN THIS RESEARCH STUDY, AND HOW LONG WILL IT TAKE?

You will be asked to take part in a program for family caregivers of stroke survivors. Caregivers will be randomized (like flipping a coin) to either the TASK III group or to the ISR group. Both groups will have **9 calls with a nurse.** Each call will last about 20-45 minutes based on your needs. You can choose to have these calls by telephone or by videoconference.

- If you are in the TASK III group, you will receive a TASK III Resource Guide. The nurse will help you: a) assess your needs and concerns; b) build your skills as a caregiver; and c) refer you to community resources.
- If you are in the ISR group, you will receive a guide about family caregiving from the American Heart Association. The nurse will provide: a) information; b) support; and c) referral to community resources.

You will also receive: a) a pedometer to track your daily steps; and b) an Amazon Fire Tablet to use in the TASK III or ISR program.

To see how our well programs work, you will be asked to take part in **5 data collection interviews:**

- one at the beginning of the program
- one at 8 weeks
- one at 12 weeks (at the end of the program)
- one at 6 months
- one at 1 year

The first interview will last about 60 minutes. The other interviews will last about 45 minutes.
You can choose to have these interviews by telephone or by videoconference.
You will be asked about:

- your age, gender, race, and your background
- the care that you provide for the stroke survivor
- your health and well-being

ARE THERE ANY RISKS TO BEING IN THIS RESEARCH STUDY?

Some questions may make you feel uncomfortable. You can refuse to answer any questions that you don't want to answer. The risk is not expected to be more than you would have in daily life.

ARE THERE ANY BENEFITS FROM BEING IN THIS RESEARCH STUDY?

You will probably not get any benefit from taking part in this study. But, being in this study may help caregivers better understand their needs.

WHAT WILL YOU GET BECAUSE OF BEING IN THIS RESEARCH STUDY?

You will not be paid to take part in this study. You will be able to keep the pedometer, Amazon Fire tablet, and the TASK III or ISR materials given to you during the study.

University of CINCINNATI

DO YOU HAVE CHOICES ABOUT TAKING PART IN THIS RESEARCH STUDY?

If you do not want to take part in this research study you may simply not participate. Your participation in this study is completely voluntary. You may withdraw at any time.

HOW WILL YOUR RESEARCH INFORMATION BE KEPT CONFIDENTIAL?

Calls with you will be made from a private location. We cannot promise that information will be private if it is sent by the internet, email, texting, online videoconferencing, website, or other electronic methods. The data collection interviews and TASK III and ISR calls with the nurse will be audiotaped. Audiotapes will be erased once they are reviewed for training purposes. Some audiotapes will be transcribed then erased. Your name will not be used on any typed transcripts or data collection forms. A study ID number will be used instead of your name. Your name and contact information will be stored separately from the transcripts and research forms. All data will be kept on: a) a password-protected computer; b) password-protected secure REDCap; c) OneDrive; d) research drive at the University of Cincinnati; or e) in locked file cabinets. Within 3 years after the study is over, all research data will be de-identified. Information that could identify you will be removed. The study data could then be used for future research studies. This may be done in the future without asking you.

Your information will be kept confidential, unless authorities have to be notified about abuse or immediate harm that may come to you or others. If you or the stroke survivor have a health issue, you will be encouraged to contact a healthcare provider. In an emergency, a healthcare provider or the emergency medical system may be contacted on your behalf. Agents of the University of Cincinnati and the National Institute of Nursing Research (NINR) may inspect study records for audit or quality assurance purposes. A description of this clinical trial will be available on ClinicalTrials.gov, as required by U.S. Law. This website will not include information that can identify you. At most, the website will include a summary of the results. You can search this website at any time. The data from this research study may be published; but you will not be identified by name.

WHAT ARE YOUR LEGAL RIGHTS IN THIS RESEARCH STUDY?

Nothing in this consent form waives any legal rights you may have. This consent form also does not release the investigator, the National Institute of Nursing Research (NINR), the institution, or its agents from liability for negligence.

WHAT IF YOU HAVE QUESTIONS ABOUT THIS RESEARCH STUDY?

If you have any questions or concerns about this research study, you should contact Dr. Tamilyn Bakas at (513) 558-2254 or by email Tamilyn.bakas@uc.edu.

The UC Institutional Review Board reviews all research projects that involve human participants to be sure the rights and welfare of participants are protected.

University of CINCINNATI

If you have questions about your rights as a participant, complaints and/or suggestions about the study, you may contact the UC IRB at (513) 558-5259. Or, you may call the UC Research Compliance Hotline at (800) 889-1547, or email the IRB office at irb@ucmail.uc.edu.

DO YOU HAVE TO TAKE PART IN THIS RESEARCH STUDY?

No one has to be in this research study. Refusing to take part will NOT cause any penalty or loss of benefits that you would otherwise have. You may skip any questions that you don't want to answer. You may start and then change your mind and stop at any time. To stop being in the study, you should tell Dr. Tamilyn Bakas at (513) 558-2254 or by email Tamilyn.bakas@uc.edu.

BY TAKING PART IN THE STUDY ACTIVITIES YOU INDICATE YOUR CONSENT FOR YOUR ANSWERS TO BE USED IN THIS RESEARCH STUDY

PLEASE KEEP THIS INFORMATION SHEET FOR YOUR REFERENCE.

>>>>> ***SEE NEXT PAGE FOR ADDITIONAL QUESTION*** >>>>>

University of CINCINNATI

Are you willing to be contacted about opportunities to participate in future studies?

_______ No

_______ Yes:

Name: ____

Address: ____

City, State, Zip Code: ___________________________________

Email address(s): _______________________________________

Phone number(s): _______________________________________ Can we text you? _______

What is the best day of the week to reach you?
___Monday
___Tuesday
___Wednesday
___Thursday
___Friday
___Saturday
___Sunday

What is the best time of the day to reach you?
___Morning (9AM-11AM)
___Afternoon (12N-5PM)
___Evening (6PM-8PM)

Thank you.
