# Supplementary material for: Examining the Efficacy of the Telehealth Assessment and Skill-Building Kit (TASK III) Intervention for Stroke Caregivers: Protocol for a Randomized Controlled Clinical Trial
Source: JMIR Res Protoc. 2025 Mar 25;14:e67219. doi: 10.2196/67219 (PMC11979539; doi:10.2196/67219)
Supplement: Multimedia Appendix 3 [file resprot_v14i1e67219_app3.pdf]

Screenshots of the TASK III Website located at: https://task3web.com

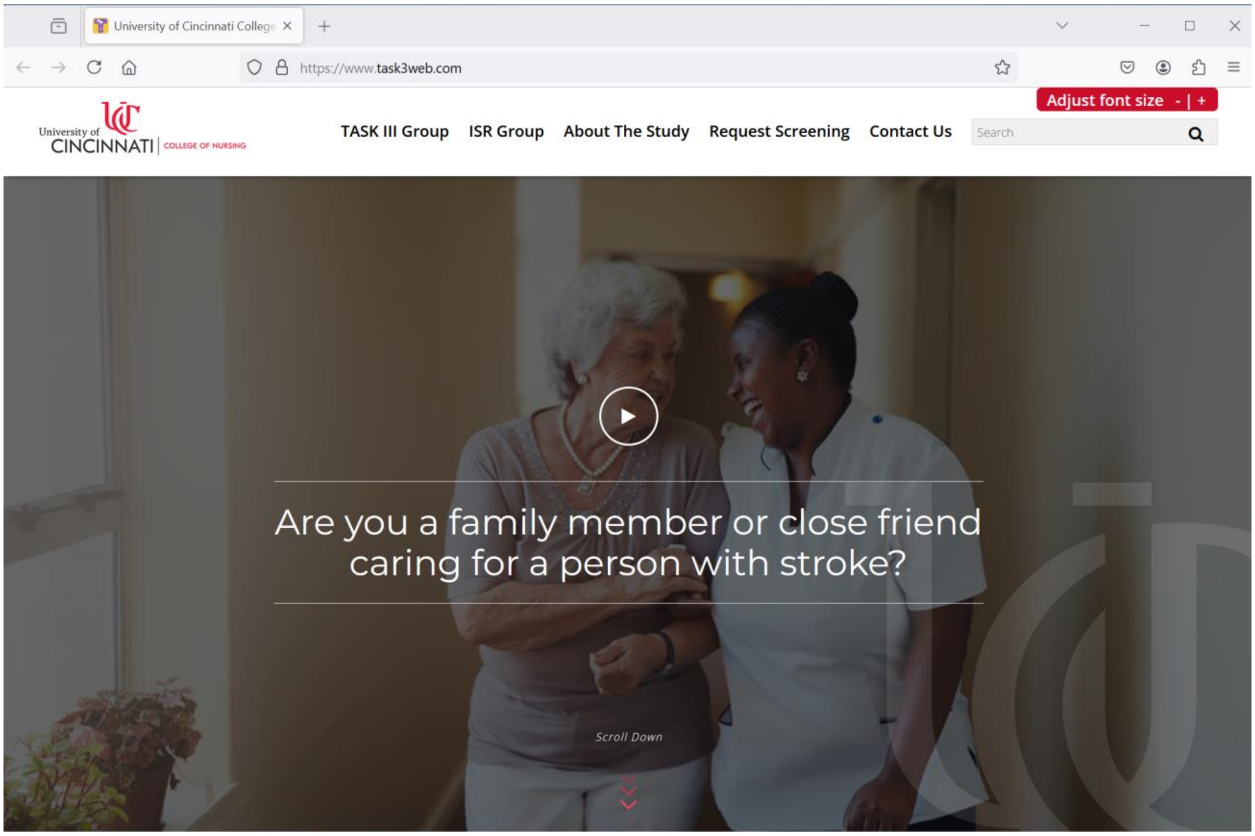

Talk with our nurses about providing care for your loved one, while also taking care of yourself as a caregiver.
Let us tell you about our study, designed to help family caregivers of stroke survivors.

Telehealth Assessment and Skill-Building Intervention for Stroke Caregivers (TASK III)

The purpose of this study is to try out a program for family caregivers of stroke survivors.
The program is called the Telehealth Assessment and Skill-Building Kit (TASK III).
The TASK III program will be compared with an Information Support and Referral (ISR) program.

Learn More ›

Request a Free Screening

Click here if you want to be contacted for a free screening to see if our study is right for you.

Free Screening ›

Telehealth Assessment and Skill-Building Kit (TASK III) Group

Login here if you have been assigned to our TASK III Group.

Login ›

Information Support and Referral (ISR) Group

Login here if you have been assigned to our ISR group.

Login ›

> "Comment from a Stroke Family Caregiver… Well of course you're overwhelmed. You don't know which way to go from the beginning. They were able to **steer me in the right way** and tell me about resources I had available."

About The Study

About The Study

Click here to learn more about our study, and to see if our study is right for you.

Learn More ›

Researchers & Staff

Meet our Project Manager, Nurses, Data Collectors, and Technicians.

Learn More ›

Funding

Our study is locally and nationally funded. Click here to see our funding sources.

Learn More ›

Key Stroke Family Caregiver Sites

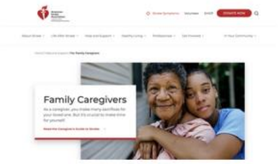

For Stroke Family Caregivers – American Stroke Association

Caregivers and family members play a critical role in the post-stroke recovery process.

Go To Website ›

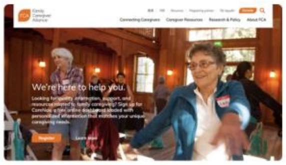

Family Caregiver Alliance

Family Caregiver Alliance supports and sustains the important work of families nationwide caring for adult loved ones with chronic, disabling...

Go To Website ›

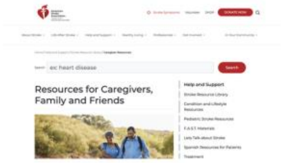

For Stroke Resources – American Stroke Association

These resources are gathered to help you navigate all aspects of how the stroke has impacted you and your survivor.

Go To Website ›

Interested in Participating?

Telehealth Assessment and Skill-Building Intervention for Stroke Caregivers (TASK III)

Contact us to learn more about our study and to request a free screening to see if our study is right for you.

Learn More ›

Free Screening ›

Interested in Learning More?

Contact us to learn more about our Research Study.

Contact Us ›

©2024 University of Cincinnati College of Nursing. All Rights Reserved. | Privacy Policy | Site Info | Site Map
