# Supplementary material for: Examining the Efficacy of the Telehealth Assessment and Skill-Building Kit (TASK III) Intervention for Stroke Caregivers: Protocol for a Randomized Controlled Clinical Trial
Source: JMIR Res Protoc. 2025 Mar 25;14:e67219. doi: 10.2196/67219 (PMC11979539; doi:10.2196/67219)
Supplement: Multimedia Appendix 4 [file resprot_v14i1e67219_app4.pdf]

Measures		Measure description	Time frame
**Primary outcome**			
	Life changes	Life changes (ie, changes in social functioning, subjective well-being, and physical health because of providing care) are measured by the BCOS[a] [7], which consists of 15 items rated on a response scale ranging from –3 (changed for the worst) to +3 (changed for the best). Items are recoded (–3=1, –2=2, –1=3, 0=4, 1=5, 2=6, and 3=7) so that positive numbers can be obtained for analysis. The recoded responses to the 15 items are summed for a total score with a possible range of 15 to 105. Higher scores indicate more positive life changes as a result of providing care. Strong psychometric properties have been reported [7-10,31,34].	• Baseline to 8 weeks (short term); baseline to 12, 24, and 52 weeks (long term)
**Secondary outcomes**			
	Depressive symptoms	• PHQ-9[b] Depression Scale [63,64] consists of 9 items rated on a scale from 0=not at all to 3=nearly every day. Higher scores reflect more depressive symptoms.	• Baseline to 8 weeks (short term); baseline to 12, 24, and 52 weeks (long term)
	Other symptoms	• Visual numeric symptoms are used in the Chronic Disease Self-Management Program study on a scale from 0 to 10 (stress, fatigue, sleep, pain, and shortness of breath) [65]. Higher scores indicate higher severity of the symptom.	• Baseline to 8 weeks (short term); baseline to 12, 24, and 52 weeks (long term)
	Unhealthy days	• Unhealthy days consist of two items that are used to measure the number of days in the past 30 days when physical and mental health have not been good [66,67]. Items are totalled and capped at 30 days for a total score, with analysis conducted at the item level as well.	• Baseline to 8 weeks (short term); baseline to 12, 24, and 52 weeks (long term)
	Self-management steps	• A pedometer is used to measure the number of steps per day	• Baseline to 8 weeks (short term); baseline to

			12, 24, and 52 weeks (long term)
	Exercise self-management	• Physical Activity Scale for Elderly [68-70] consists of 10 items measuring physical activity. The total score is computed by multiplying the amount of time spent in each activity (hr/wk) or participation (yes or no) in an activity by empirically derived item weights and summing overall activities. The score can be stratified in tertiles: 0-40 (sedentary), 41-90 (light physical activity), and >90 (moderate to intense activity).	• Baseline to 8 weeks (short term); baseline to 12, 24, and 52 weeks (long term)
	Diet self-management	• Dietary Screener Questionnaire [71-73] consists of 30 items measuring diet intake. Scores provide predicted intake of fruits, vegetables, added sugars, whole grains, fiber, calcium, and dairy.	• Baseline to 8 weeks (short term); baseline to 12, 24, and 52 weeks (long term)
	Health care use	• Hospitalizations; emergency room visits; office visits (medical, dental, and vision); immunizations; and screening are self-reported in the past 3 months [65].	• Baseline to 8 weeks (short term); baseline to 12, 24, and 52 weeks (long term)
**Mediators**			
	Task difficulty	• Oberst Caregiving Burden Scale has 15 caregiving tasks rated based on the level of difficulty [8,74], ranging from –3 (extremely difficult) to +3 (extremely easy). The items are recoded (–3=1, –2=2, –1=3, 0=4, 1=5, 2=6, and 3=7) so that positive numbers can be obtained for analysis. The recoded responses to the 15 items are summed for a total score with a possible range of 15 to 105. Lower scores indicate more difficulty with caregiving tasks.	• Baseline to 8 weeks (short term); baseline to 12, 24, and 52 weeks (long term)
	Threat appraisal	• Appraisal of Caregiving Threat Subscale [74, 75] consists of 12 items rated on a scale from 1=strongly disagree to 5=strongly agree. Higher scores indicate	• Baseline to 8 weeks (short term); baseline to

		higher threat appraisal. Items include caregiver self-efficacy, for example, "I worry that I will not be able to help the patient in the future."	12, 24, and 52 weeks (long term)
	Exercise self-efficacy	• The Self-Efficacy Exercise Scale [76,77] consists of 10 items rated on a scale from 0=not at all confident to 10=extremely confident. Higher scores indicate greater self-efficacy in exercise.	• Baseline to 8 weeks (short term); baseline to 12, 24, and 52 weeks (long term)
	Diet self-efficacy	• The Self-Efficacy Diet Scale [76,77] consists of 10 items rated on a scale from 0=not at all confident to 10=extremely confident. Higher scores indicate greater self-efficacy in maintaining a healthy diet.	• Baseline to 8 weeks (short term); baseline to 12, 24, and 52 weeks (long term)
**Program evaluation**			
	Satisfaction	• CSS[c] [32] measures the usefulness, ease of use, and acceptability of the TASK III[d] and ISR[e] interventions, consisting of 9 items rated on a scale from 1=strongly disagree to 5=strongly agree. Higher scores indicate more positive program outcomes.	• 12 weeks
	Technology	• Caregiver Technology Evaluation Scale consists of 8 items rated on a scale from 1=strongly disagree to 5=strongly agree. Higher scores indicate more positive program outcomes.	• 12 weeks
**Sample characteristics**			
	Caregiver and survivor demographics	• The caregiver demographic data form includes items such as age, sex, race, ethnicity, education, income, type of relationship to survivor, marital status, and living arrangement. Survivor demographic data form includes age, sex, race, ethnicity, hospital days, and days discharged. Comorbidities for both caregiver and survivor include the Chronic Conditions Index [78].	• Baseline
	Caregiver and survivor	• Cognitive Status Scale [79]<br>• Proxy Modified Rankin Scale [81,82]	• Baseline

	cognition or impairment	• Stroke Impact Scale Proxy [83-85] version consists of 16 items for the overall physical domain of stroke (strength, hand function, activities of daily living or instrumental activities of daily living, and mobility) based on the level of difficulty.<br>• A 6-item screener for cognitive impairment [86] is used to screen the eligibility of the caregiver to participate.	• Baseline and 8, 12, 24, and 52 weeks<br>• Screening
	Other social determinants of health	• Social determinants of health defined by Adler and Stead [48] are used with items from the PhenX toolkit [49]. It includes financial resource strain, nicotine and alcohol use or exposure, social connections or isolation, neighborhood characteristics, food insecurity, and access to health services. The measurement provides a more detailed sample description and is useful to train caregivers to select skill-building strategies to address needs and concerns in the context of their own social determinants of health.	• Baseline

[a]BCOS: Bakas Caregiving Outcomes Scale.

[b]PHQ-9: Patient Health Questionnaire-9.

[c]CSS: Caregiver Satisfaction Scale.

[d]TASK III: Telehealth Assessment and Skill-Building Kit.

[e]ISR: information, support, and referral.
