# Supplementary material for: Examining the Efficacy of the Telehealth Assessment and Skill-Building Kit (TASK III) Intervention for Stroke Caregivers: Protocol for a Randomized Controlled Clinical Trial
Source: JMIR Res Protoc. 2025 Mar 25;14:e67219. doi: 10.2196/67219 (PMC11979539; doi:10.2196/67219)
Supplement: Multimedia Appendix 5 [file resprot_v14i1e67219_app5.pdf]

1R01NR020184-01 BAKAS, TAMILYN

INCLUSION ACROSS THE LIFESPAN PLAN UNACCEPTABLE

RESUME AND SUMMARY OF DISCUSSION: This application proposes to test the short- and long-term efficacy of a nurse-led Telephone Assessment and Skill-Building Kit (TASK) intervention addressing the needs of family caregivers managing the care for stroke survivors. An intervention targeting family caregivers of people living with stroke fills a significant need for providing the problem solving, stress management, and goal setting skills necessary when assuming the caregiver role. The research team is well qualified with a prior history of collaboration. The intervention includes innovative elements such as the caregiver choice of how they want to access the resource guide and how they wish to interact with the nurse. The approach is grounded in preliminary data including the feedback from caregivers themselves and supports the content validity, treatment fidelity, caregiver satisfaction, and efficacy of the intervention. The screening and recruitment procedures are detailed and ensure the ability to reach the targeted recruitment goals. Grounding the intervention in self-management and stress management strategies informed by Lazarus' transactional approach and the team's empirically derived conceptual model is a strength. Reviewers only discussed minor and addressable weaknesses, notably that there are aspects of the attention control condition that overlap with TASK and could limit clear distinction between the two groups and it is unclear how this risk will be monitored. Overall, this is a highly significant and innovative application that will have a high impact on supporting family caregivers in caring for themselves as they manage care for people living with stroke.

DESCRIPTION (provided by applicant): Stroke is a leading cause of serious, long-term disability, and has a very sudden onset. Families are often thrust into providing care without sufficient training from health care providers, having to learn on their own to provide care. Studies show that caregiving without proper training can be detrimental to caregiver's physical and mental health, which can impede survivor rehabilitation and lead to institutionalization and higher societal costs. Unlike existing stroke caregiver interventions, which require costly face-to-face interactions, and that focus primarily on the survivor's care, the nurse-led Telephone Assessment and Skill-Building Kit (TASK II) is delivered completely by telephone, and empowers caregivers to address both their own and the survivor's needs using innovative skill-building strategies. Aligned with current patient and caregiver guidelines, TASK II demonstrated evidence of content validity, treatment fidelity, caregiver satisfaction, and efficacy for reducing caregiver depressive symptoms; however, TASK II revealed a need for a stronger focus on self-management (SM) strategies to improve caregiver symptoms and health, along with enhanced use of other telehealth modes of delivery. In an NINR-funded R21, the Telehealth Assessment and Skill-building Kit (TASK III) was optimized through the innovative leveraging of technologies and SM strategies to improve stroke family caregiver symptoms and health. A new goal setting tip sheet promotes caregivers' self-management of their own symptoms and health through the use of novel skill-building strategies. Caregivers now choose how they want to access the TASK III Resource Guide (mailed hard copy, eBook, USB drive, or interactive website (https://www.task3web.com/) and how they would like to interact with the nurse (telephone, FaceTime, or online videoconferencing). Preliminary TASK III data provided evidence for feasibility of recruitment, retention, treatment fidelity, high satisfaction, and positive data trends in 74 stroke family caregivers randomized to TASK III (n=36) or to an Information, Support, and Referral (ISR) group (n=38). The purpose of the proposed study is to test short-term (immediately post-intervention at 8 weeks) and long-term (12, 24, and 52 weeks) efficacy of the TASK III intervention, compared with the ISR group, in 296 stroke caregivers. The primary outcome is caregiver life changes (i.e., physical health, physical functioning, emotional well-being, general health) as a result of providing care. Secondary outcomes include depressive symptoms (in caregivers with mild to severe depressive symptoms), other symptoms (stress, fatigue, sleep, pain, shortness of breath), unhealthy days, SM of diet/exercise, and self-reported healthcare utilization. Theoretically-based mediators include task difficulty, threat appraisal, and self-efficacy. Program evaluation outcomes (satisfaction, technology ratings) will also be analyzed. If TASK III is shown to be efficacious

in the proposed randomized controlled clinical trial, our next goal will be to translate TASK III into ongoing stroke systems of care; and to adapt it for use among caregivers with other debilitating/chronic conditions providing a tremendous public health impact.

PUBLIC HEALTH RELEVANCE: Caring for a family member after a stroke can be very difficult and worsen the physical and mental health of untrained caregivers. The TASK III intervention is a unique, comprehensive caregiver intervention program that enables caregivers to develop the necessary skills to manage care for the survivor, while also taking care of their own health.

CRITIQUE 1

Significance: 2
Investigator(s): 1
Innovation: 2
Approach: 3
Environment: 1

Overall Impact: This application proposes an RCT to test the short and long-term efficacy of the Telephone Assessment and Skill Building Kit (TASK III) intervention in 296 stroke caregivers. The primary outcome is caregiver physical health (physical functioning, emotional well-being, and general health). Secondary outcomes include depressive symptoms stress, fatigue, sleep, pain, shortness of breath, unhealthy days, and self-reported healthcare utilization. Theoretically-based mediators include task difficulty, threat appraisal, and self-efficacy. Program evaluation outcomes will also be analyzed. The project is innovative in tailoring delivery to caregiver preference with traditional telephone and mailed TASK III Resource Guide or using videoconferencing technologies with an electronic version of the TASK III Resource Guide. The investigator is a productive senior investigator in family caregiving. Other score driving strengths in this application including the strong collaborative/ interdisciplinary team, the exemplary environment , the use of RTC design to evaluate the effective's using clinically meaningful outcomes. Score driving weaknesses include the missed opportunity to collect data to inform future implementation and scaling, and missing details in approach including blinding of study team, and crossover of elements of intervention in the attention control group. On balance of the many strengths and few weaknesses, the application is scored at moderate high impact.

1. Significance:

Strengths

- This proposed project is addressing an important area: Family members of persons with stroke are often lacking practical problem solving skills, stress management skills and goal setting skills required when assuming the caregiving role.
- Task III content is aligned with AHA recommendations and other published guidelines and comes with a strong track record of generating and publishing evidence of its feasibility, acceptability and preliminary efficacy.
- Addresses a gap in that there are few stroke caregiver intervention studies that use telehealth technologies to reach caregivers living in distant settings

Weaknesses

- Important missed opportunity to collect information that would inform future implementation in stroke systems of care limits potential impact of findings

2. Investigator(s):

Strengths

- The PI is a senior investigator with a strong background in family caregiving research. This R01 builds sequentially on her K01 and R21 to develop TASK program. She has assembled a strong interdisciplinary research team with complementary expertise in nursing, medicine, biostatistics, information technology, bioinformatics, physical therapy, dietary sciences, and social work. The team has worked together in prior studies of the TASK program

Weaknesses

- None noted

3. Innovation:

Strengths

- Novel elements include caregiver choice of how they want to access the TASK III Resource Guide (mailed hard copy, eBook, USB drive, or interactive website and how they would like to interact with the nurse (telephone, FaceTime, or online videoconferencing).

Weaknesses

- N/A

4. Approach:

Strengths

- Strong preliminary evidence in support of TASK III demonstrated content validity, treatment fidelity, caregiver satisfaction, and efficacy.
- Adapted TASK II to include a stronger focus on self-management strategies and with enhanced use of other telehealth modes of delivery
- Well detailed screening and recruitment procedures ensure ability to reach targeted recruitment goals.
- Detailed randomization procedure
- Theoretically grounded content in self-management and stress management strategies informed by Lazarus' transactional approach, team's empirically derived conceptual model, technology acceptance, and concepts from self-efficacy theory
- Evidence of scientific rigor via attention to fidelity, use of reliable/valid instruments, and use of randomized trial design with attention control condition
- Sex as biological factor addressed

Weaknesses

- Unclear how blinding of study team (data collectors) will be maintained
- No detail on training of interventionist (nurses)
- Aspects of the attention control condition overlap with TASK ("information, support") limit clear distinction between two groups. Unclear how this risk will be monitored

5. Environment:

Strengths

- Strong resources at University of Cincinnati, College of Nursing (Center for Academic Technologies & Educational Resources University of Cincinnati Neuroscience Institute:
- Clinical resources at Greater Cincinnati/Northern Kentucky Stroke Team University of Cincinnati Medical Center with letters of support from UC Health/UC Health West Chester Hospital at the University of Cincinnati, Merc

Weaknesses

- None noted

Study Timeline:

Strengths

- Sufficiently detailed timeline

Weaknesses

- None noted by Reviewer

Protections for Human Subjects

Acceptable Risks and/or Adequate Protections

Data and Safety Monitoring Plan (Applicable for Clinical Trials Only):

Acceptable

Inclusion Plans

- Sex/Gender: Distribution justified scientifically
- Race/Ethnicity: Distribution justified scientifically
- For NIH-Defined Phase III trials, Plans for valid design and analysis: Scientifically acceptable
- Inclusion/Exclusion Based on Age: Distribution justified scientifically

Vertebrate Animals

Not Applicable (No Vertebrate Animals)

Biohazards

Not Applicable (No Biohazards)

Resource Sharing Plans

Acceptable

Authentication of Key Biological and/or Chemical Resources

Not Applicable (No Relevant Resources)

Budget and Period of Support

Recommend as Requested

CRITIQUE 2

Significance: 1
Investigator(s): 1
Innovation: 2
Approach: 2
Environment: 1

Overall Impact: This proposal addresses a critical area of need, that of caregiver skills and training - particularly in person post stroke. The proposal uses technology to provide a nurse led assessment and skill building program. This proposal is strengthened by preliminary data from an earlier R21 and its grounding in theory (self-management). The RCT is well designed and incorporates critical aspects of self-management and caregiver well-being. Given the issue raised from supports during the pandemic, the application of this protocol to online using telehealth is a strength

1. Significance:

Strengths

- Stroke is extremely debilitating and happens quickly. The proposal well situates the need for caregiver training for an illness that comes on fast and has no existing preparation.
- Well placed in the context of Covid, and the need to continue focusing on support programs using non face to face and integrated technology formats, including phone for those without broadband or computers in the home
- Previous work by the authors establishes the clear need for a targeted intervention/support/skill program – as caregiving can be extremely burdensome especially given the lack of clear trainings and support across caregiver populations

Weaknesses

- None noted

2. Investigator(s):

Strengths

- PI has long history of research in this area and has received K award and R21 to support earlier work. Well supported and trained for this project
- Solid interdisciplinary team, well suited for this type of project
- Numerous publications and concurrent projects together as a team

Weaknesses

- Consultants' roles are unclear, particularly the social worker. The PI and primary team will engage the caregivers, thus unclear where the social worker comes in

3. Innovation:

Strengths

- The provision of support for caregivers is not in and of itself innovative, however the inclusion of multiple tele modalities and the broad inclusion of assessment tools by the caregivers is somewhat innovative

Weaknesses

- The provision of support for caregivers is not in and of itself innovative

4. Approach:

Strengths

- Lots of preliminary data (including feasibility) supporting this proposal, including previously received R21 and K award, and most importantly feedback from the caregivers themselves
- Inclusion of adults ranging from 21 – 80 is a strength of the proposal
- Measures are well validated and used previously in preliminary work for the proposal

Weaknesses

- Only group specifically discussed was African American, others will be invited, but this brings to question accessibility for those where English is not their first language. Unclear how this will be handled as English only is an eligibility criteria
- It is unclear how long each session with the caregiver takes – how long for each call?
- There are no measures for caregiving self-efficacy, which is surprising given the clear connection between task self-efficacy and overall well-being, regardless of disease or care type

5. Environment:

Strengths

- Large collaboration with excellent existing facilities.
- Numerous technology resources that are clearly targeted to this type of telehealth project
- High level research university and collaborators

Weaknesses

- None noted

Study Timeline:

Strengths

- Clearly able to start ASAP given the previous work and solid collaborations

Weaknesses

- None noted

Protections for Human Subjects

Acceptable Risks and/or Adequate Protections

Data and Safety Monitoring Plan (Applicable for Clinical Trials Only):

Acceptable

- o would be useful to have a family caregiver as part of the plan.

Inclusion Plans

- Sex/Gender: Distribution justified scientifically
- Race/Ethnicity: Distribution justified scientifically
- For NIH-Defined Phase III trials, Plans for valid design and analysis: Scientifically acceptable
- Inclusion/Exclusion Based on Age: Distribution not justified scientifically
- The lack of inclusion of youth caregivers is not based in data and is often quite appropriate. Caregiving under the age of 21 are quite common and should be included in caregiving supports and interventions

Vertebrate Animals

Not Applicable (No Vertebrate Animals)

Biohazards

Not Applicable (No Biohazards)

Resource Sharing Plans

Authentication of Key Biological and/or Chemical Resources

Not Applicable (No Relevant Resources)

Budget and Period of Support

Recommended budget modifications or possible overlap identified:

CRITIQUE 3

Significance: 1
Investigator(s): 1
Innovation: 1
Approach: 2
Environment: 1

Overall Impact: The purpose of the proposed study is to test short-term and long-term efficacy of the TASK III intervention a self-management intervention for caregivers of stroke patients that is an extension of the TASK II intervention. There is strong prior research supporting this proposal and the efficacy of TASK III. There is a strong investigative led by an experienced PI. This proposal addresses a significant problem of supporting stroke caregivers with innovative developments in the TASK III iteration (including multimodal approaches to receiving the intervention). The experimental strategy is sound and likely reproducible. This proposal is likely to have a high level of impact.

1. Significance:

Strengths

- This is a highly significant problem given the burden of stroke caregiving.
- There is strong prior research supporting the premise of this proposal, the intervention efficacy, and the focus on self-management.

Weaknesses

- None noted.

2. Investigator(s):

Strengths

- Verry strong PI with a record of successful completion of rigorous proposals leading up to the TASKIII iteration of the intervention.
- Strong multidisciplinary investigative team with complementary experience and expertise.

Weaknesses

- None noted.

3. Innovation:

Strengths

- Giving the participants options of how to engage (phone, facetime, zoom) is innovative and likely to enhance success of the proposal.

Weaknesses

- None noted.

4. Approach:

Strengths

- This proposal clearly overcomes limitations of prior research through the large rigorous RCT with multimodal video and telehealth options.
- The design is informed by prior research including the PI’s previous R21 and R01, which enhances confidence in the success of the proposal. There is strong data presented to support the intervention would be efficacious.
- Data provided on recruitment for previous studies enhances confidence in feasibility of the proposal.
- The approach is well-reasoned with sound rigorous experimental strategy including appropriate control group, plans for treatment fidelity tracking and missing data.
- Appropriate consideration of sex as a biological variable in the analyses.

Weaknesses

- It’s not clear why the power analysis is done based on depression, when that is the secondary outcomes.

5. Environment:

Strengths

- The team has access to resources needed to successfully complete the project.

Weaknesses
- None noted.

Study Timeline:

Strengths
- Supported by prior data from their r21 feasibility trial.

Weaknesses
- None noted.

Protections for Human Subjects

Acceptable Risks and/or Adequate Protections

Data and Safety Monitoring Plan (Applicable for Clinical Trials Only):

Acceptable

Inclusion Plans
- Sex/Gender: Distribution justified scientifically
- Race/Ethnicity: Distribution justified scientifically
- For NIH-Defined Phase III trials, Plans for valid design and analysis:
- Inclusion/Exclusion Based on Age: Distribution justified scientifically

Vertebrate Animals

Not Applicable (No Vertebrate Animals)

Biohazards

Not Applicable (No Biohazards)

Resource Sharing Plans

Not Applicable (No Relevant Resources)

Authentication of Key Biological and/or Chemical Resources

Not Applicable (No Relevant Resources)

Budget and Period of Support

Recommend as Requested

THE FOLLOWING SECTIONS WERE PREPARED BY THE SCIENTIFIC REVIEW OFFICER TO SUMMARIZE THE OUTCOME OF DISCUSSIONS OF THE REVIEW COMMITTEE, OR REVIEWERS' WRITTEN CRITIQUES, ON THE FOLLOWING ISSUES:

PROTECTION OF HUMAN SUBJECTS: ACCEPTABLE

INCLUSION OF WOMEN PLAN: ACCEPTABLE

INCLUSION OF MINORITIES PLAN: ACCEPTABLE

INCLUSION ACROSS THE LIFESPAN PLAN: UNACCEPTABLE

- The lack of inclusion of youth caregivers is not based in data and is often quite appropriate. Caregiving under the age of 21 are quite common and should be included in caregiving supports and interventions

COMMITTEE BUDGET RECOMMENDATIONS: The budget was recommended as requested.

Footnotes for 1 R01 NR020184-01; PI Name: BAKAS, TAMILYN

NIH has modified its policy regarding the receipt of resubmissions (amended applications).See Guide Notice NOT-OD-18-197 at https://grants.nih.gov/grants/guide/notice-files/NOT-OD-18-197.html. The impact/priority score is calculated after discussion of an application by averaging the overall scores (1-9) given by all voting reviewers on the committee and multiplying by 10. The criterion scores are submitted prior to the meeting by the individual reviewers assigned to an application, and are not discussed specifically at the review meeting or calculated into the overall impact score. Some applications also receive a percentile ranking. For details on the review process, see http://grants.nih.gov/grants/peer_review_process.htm#scoring.
